# miR-137 and miR-122, two outer subventricular zone non-coding RNAs, regulate basal progenitor expansion and neuronal differentiation

**DOI:** 10.1016/j.celrep.2022.110381

**Published:** 2022-02-15

**Authors:** Ugo Tomasello, Esther Klingler, Mathieu Niquille, Nandkishor Mule, Antonio J. Santinha, Laura de Vevey, Julien Prados, Randall J. Platt, Victor Borrell, Denis Jabaudon, Alexandre Dayer

**Affiliations:** 1 Department of Basic Neurosciences, University of Geneva, 1205 Geneva, Switzerland; 2 Department of Psychiatry, Geneva University Hospital, 1205 Geneva, Switzerland; 3 Clinic of Neurology, Geneva University Hospital, 1205 Geneva, Switzerland; 4 Instituto de Neurociencias, Consejo Superior de Investigaciones Científicas and Universidad Miguel Hernández, Sant Joan d'Alacant, 03550 Alacant, Spain; 5 Department of Biosystems Science and Engineering, ETH Zurich, 4058 Basel, Switzerland

**Keywords:** evolution, microRNA, neurogenesis, basal progenitors, cortex, neuronal maturation

## Abstract

Cortical expansion in primate brains relies on enlargement of germinal zones during a prolonged developmental period. Although most mammals have two cortical germinal zones, the ventricular zone (VZ) and subventricular zone (SVZ), gyrencephalic species display an additional germinal zone, the outer subventricular zone (oSVZ), which increases the number and diversity of neurons generated during corticogenesis. How the oSVZ emerged during evolution is poorly understood, but recent studies suggest a role for non-coding RNAs, which allow tight genetic program regulation during development. Here, using *in vivo* functional genetics, single-cell RNA sequencing, live imaging, and electrophysiology to assess progenitor and neuronal properties in mice, we identify two oSVZ-expressed microRNAs (miRNAs), miR-137 and miR-122, which regulate key cellular features of cortical expansion. miR-137 promotes basal progenitor self-replication and superficial layer neuron fate, whereas miR-122 decreases the pace of neuronal differentiation. These findings support a cell-type-specific role of miRNA-mediated gene expression in cortical expansion.

## Highlights

• oSVZ-expressed microRNAs 137 and 122 promote superficial layer identity of neurons
• miR-137 promotes basal progenitor proliferation and layer 2/3 neuron generation
• miR-122 slows down neuronal differentiation pace

## Graphical abstract

**Figure:**
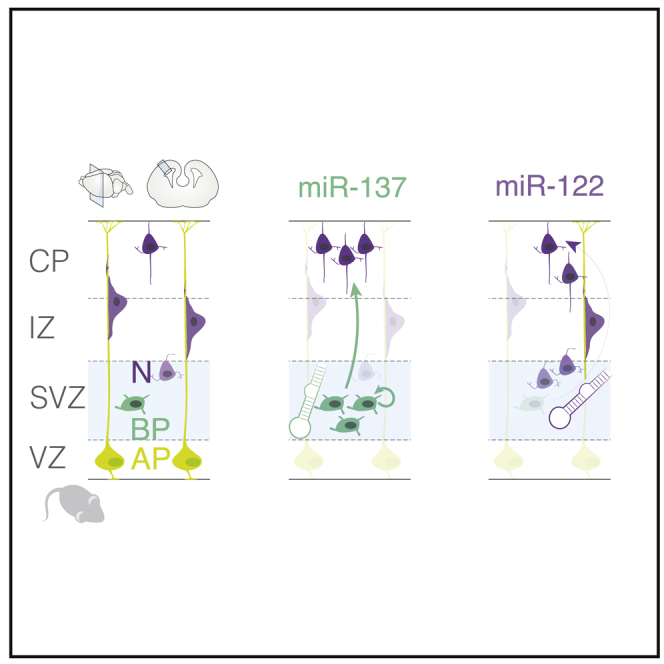

## Teaser

Tomasello et al. demonstrate the role of two microRNAs (miRNAs) during cortical expansion. miR-137 induces proliferative behavior on basal progenitors in the subventricular zone (SVZ), and miR-122 slows down excitatory neuron differentiation progression. These distinct but complementary processes amplify the generation and molecular acquisition of layer 2/3 excitatory neurons.

## Introduction

The human cortex has circumvolutions, but many species, including within primates, have a smooth cortex instead (Rakic, 2000, 2009). Adult cortical neuron numbers depend in particular on progenitor numbers during corticogenesis, on their ability to self-amplify, and on the duration of the neurogenic period. In mammals, two main types of cortical progenitors reside in distinct germinal zones: (1) the ventricular zone (VZ), hosting apical progenitors (APs; also called apical radial glia) (Malatesta et al., 2000; Noctor et al., 2001), which abuts the ventricular wall, and (2) the subventricular zone (SVZ), located more superficially, which contains basal progenitors (BPs) that amplify neuronal output from APs (Noctor et al., 2004; Haubensak et al., 2004; Miyata et al., 2004). In gyrencephalic species, BPs seem to have undergone an increase in proliferation capacity, causing SVZ thickening and emergence of anatomical subcompartments to form an “inner” SVZ (iSVZ) and “outer” SVZ (oSVZ) (Smart et al., 2002; Lukaszewicz et al., 2005; Hansen et al., 2010; Lui et al., 2011; Fietz et al., 2010; Reillo and Borrell, 2012). This expansion is particularly visible late in corticogenesis, as superficial layer neurons are being produced.

How SVZ expansion occurred during evolution is poorly understood, but fine regulatory gene expression control over the proliferation and differentiation of progenitors and neurons may have played a role (Fietz et al., 2010, 2012; Reillo et al., 2011, Reillo and Borrell, 2012). Non-coding RNAs and especially microRNAs are particularly interesting candidates in this respect because their numbers increase with evolution (Liu et al., 2013). Non-coding RNAs fine-tune various aspects of neurogenesis, allowing BP amplification in the oSVZ and, secondarily, cortical expansion, in particular via gyrus formation (Martinez-Martinez et al., 2016; Nowakowski et al., 2018; Arcila et al., 2014; Ayoub et al., 2011; Fietz et al., 2012; De Juan Romero et al., 2015; Diaz et al., 2020). Here we show that, upon overexpression of the ferret and human oSVZ-expressed microRNAs miR-137 and miR-122 in the mouse neocortex (which is normally devoid of these microRNAs), miR-137 acts to increase proliferative divisions in BPs, ultimately resulting in increased superficial layer neuron production, whereas miR-122 acts in newborn neurons to slow their differentiation pace, potentially allowing longer distances to be covered before differentiation occurs. These results reveal critical cell-type-specific roles for microRNAs in the molecular regulation of cellular properties during cortical expansion.

## Results

### miR-122 and miR-137 are expressed in the ferret cortex oSVZ during superficial layer generation

**Figure 1:**
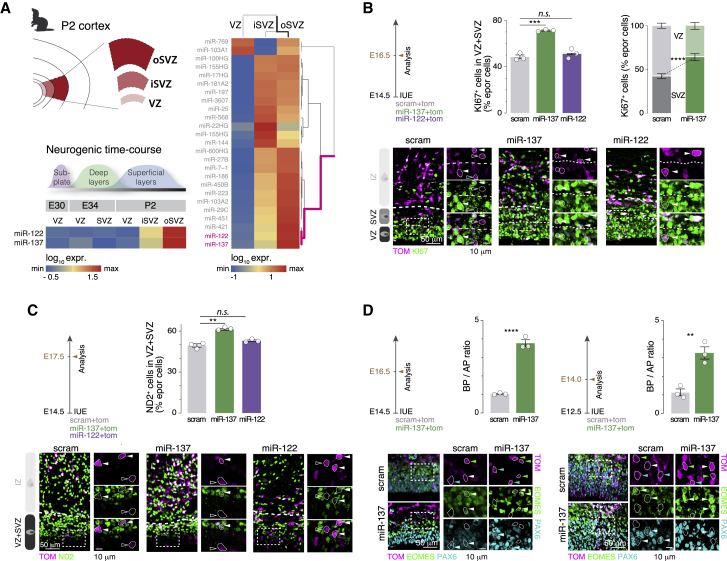
OSVZ-expressed miR-137, but not miR-122, affects cortical progenitor proliferation (A) Microarray of the ferret ventricular zone (VZ), inner SVZ (iSVZ), and outer SVZ (oSVZ) during cortex development. Data are from De Juan Romero et al. (2015) and Martinez-Martinez et al. (2016) for post-natal day 2 (P2) and embryonic day 30 (E30)/E34/P2, respectively. Top left: schematic of microdissections of the P2 cortex performed for collection of P2 VZ, iSVZ, and oSVZ. Right: expression of miRNAs in the three germinal zones on P2. miR-122 and miR-137 cluster together as most expressed in the oSVZ. Bottom left: expression of miR-137 and miR-122 in the VZ and SVZ along development. (B) Progenitors in the VZ and SVZ at E16.5 upon miR-137 or miR-122 overexpression in the E14.5 mouse cortex. Top left: experimental design. Top right: quantification of KI67^+^ electroporated cells in the VZ and SVZ (scram, miR-137, and miR-122; n = 3 each). Bottom: representative micrographs of KI67^+^ electroporated cells. Electroporated cells positive (filled arrowheads) and negative (empty arrowheads) for KI67 are highlighted. (C) Neurogenic output on E17.5 upon miR-137 or miR-122 overexpression in the E14.5 mouse cortex. Top left: experimental design. Top right: quantification of NeuroD2 (ND2)^+^ electroporated cells in the VZ and SVZ (scram, miR-137, and miR-122; n = 3 each). Bottom: representative micrographs of electroporated cells labeled for ND2. Electroporated cells positive (filled arrowheads) and negative (empty arrowheads) for ND2 are highlighted. (D) Basal progenitor (BP) versus apical progenitor (AP) ratio upon miR-137 overexpression on E14.5 (left) or E12.5 (right), addressed using immunohistochemistry against EOMES to identify BPs and PAX6 to identify APs (scram and miR-137, n = 3 each for the E16.5 and E14.0 time points). Bottom: representative micrographs of EOMES/PAX6 labeling. Green arrowheads, EOMES^+^ electroporated cells; cyan arrowheads, PAX6^+^ electroporated cells, white arrowheads, EOMES^+^/PAX6^+^. Data are represented as mean ± SEM. Biological replicates are distinguished by circles in the bar plots. One-way ANOVA (B, bottom right; C; and D, left), two-way ANOVA (B, bottom left), and unpaired t test (D, right). ^∗^p < 0.05, ^∗∗^p < 10^−2^, ^∗∗∗^p < 10^−3^, ^∗∗∗∗^p < 10^−4^.

To identify microRNAs that could regulate neurogenic programs in gyrencephalic species, we first performed a screen using RNA expression data from the three germinal compartments (VZ, iSVZ, and oSVZ) of the developing ferret cortex on postnatal day 2 (P2), when superficial layer (SL) neurons (i.e., layer 2/3 [L2/3] and L4) are being born (De Juan Romero et al., 2015; GEO: GSE60687). This analysis identified 23 expressed microRNAs, with miR-137 and miR-122 being the most strongly expressed in the oSVZ (Figure 1A, right) at the time of SL neuron generation (Figure 1A, bottom left; data from Martinez-Martinez et al., 2016; GEO: GSE63203). miR-137 is also expressed in the embryonic oSVZ of macaques (Arcila et al., 2014; miR-122 expression was not quantified), and in humans, miR-137 and miR-122 are expressed in germinal zones during SL neurogenesis (gestational weeks 19–20; Nowakowski et al., 2018; Figures S1A and S1B). In line with the lack of a distinctive oSVZ compartment in the mouse, only very low levels of both microRNAs (miRNAs) were detected at embryonic day 15.5 (E15.5) (De Pietri Tonelli et al., 2006; Figure S1E; STAR Methods), consistent with previous data obtained with microarrays (Volvert et al., 2014).

### miR-137, but not miR-122, affects cortical progenitor proliferation in mice

Based on the spatial and temporal specificity of expression of miR-137 and miR-122, we hypothesized that they could regulate key properties of oSVZ progenitors and their progenies. To test this hypothesis, we used a gain-of-function approach in mice, in which oSVZ-type progenitors are normally very rare (Shitamukai et al., 2011; Wang et al., 2011). miR-137 or miR-122 was overexpressed using *in utero* electroporation (IUE) on E14.5, when SL neuron generation is starting. We first performed a time course analysis 24, 48, and 72 h after electroporation (i.e., on E15.5, E16.5, and E17.5) to assess progenitor proliferation, using KI67 as a marker for cycling cells. We found that miR-137 expanded progenitor numbers (KI67^+^ cells) 48 h after IUE (E16.5; Figure 1B); most of these progenitors were located in the SVZ, where BPs normally reside (Figure 1B, top right). In contrast, miR-122 did not affect KI67^+^ cell numbers (Figures 1B and S1F, left). We also followed the fate of miR-137- and miR-122-overexpressing cells using NeuroD2 immunostaining to identify postmitotic neurons (Figure 1C); miR-137 increased neuronal production 72 h after IUE, but miR-122 had no such effect (Figure 1C). Using PAX6 and EOMES immunostaining to distinguish APs and BPs, we found a 3-fold increase in the BP/AP ratio following miR-137 expression (Figure 1D), whereas miR-122 did not alter the BP/AP ratio (Figure S1F, right). A similar increase in BP numbers was also observed following E12.5 electroporation, suggesting that the transcriptional networks modulated by miR-137 are accessible before SL neuron generation (Figure 1D, right). These results reveal that miR-137 overexpression increases the BP pool and subsequent neuron numbers in the mouse neocortex.

### miR-137 overexpression promotes BP generation and proliferation

**Figure 2:**
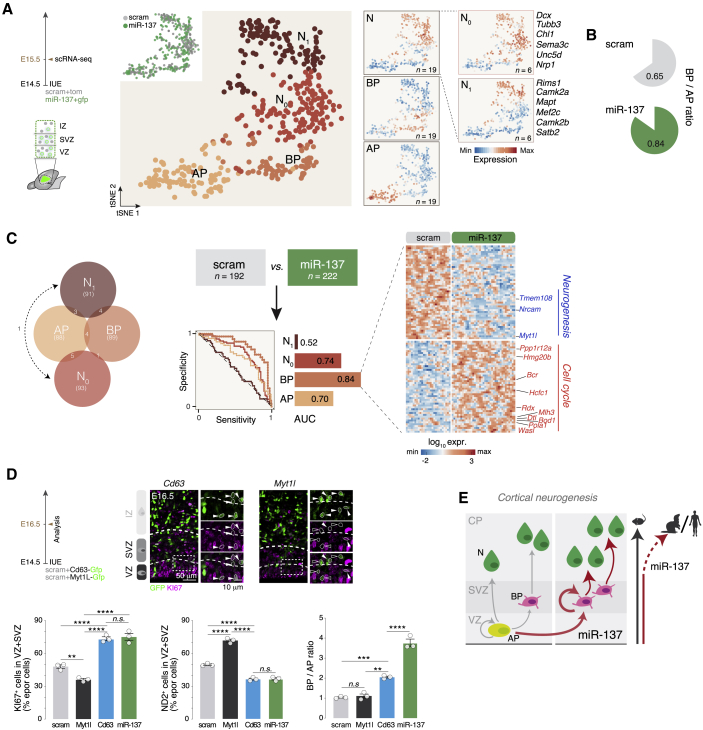
miR-137 regulates transcriptional programs promoting BP generation and proliferation (A) t-SNE representation of E15.5 scramble (scram) and miR-137 E14.5-*in utero* electroporation (IUE) scRNA-seq data reveals transcriptional organization of the cells according to their differentiation status. APs, BPs, newborn (N_0_) neurons, and differentiating neurons (N_1_) can be distinguished by their combinatorial expression of key marker genes (scram and miR-137, n = 3 each). (B) BP/AP ratio under scram and miR-137 conditions. (C) Machine learning approach to identify cell-type-specific core sets of genes classifying neurons and progenitors in miR-137 and scram conditions. Left: differentially expressed genes shared between cell types. Center: Machine learning prediction score for each cell type, shown as the area under the specificity/sensitivity curves (AUC). Note the highest score in BPs. Right: Heatmap of the top miR-137-induced and -repressed genes in BPs. (D) Progenitor and neuron numbers and BP/AP ratio upon overexpression of *Cd63* (induced by miR-137) or *Myt1l* (repressed by miR-137) on E16.5. Progenitors were identified as KI67^+^ cells, neurons as NeuroD2 (ND2)^+^ cells, and BPs and APs as EOMES^+^ and PAX6^+^ cells, respectively. Top: representative micrographs of KI67 immunohistochemistry upon *Cd63* or *Myt1l* overexpression. Bottom: quantification of KI67^+^, ND2^+^, and BP/AP ratio of electroporated cells in the VZ and SVZ (scram, Myt1l, Cd63, and miR-137; n = 3 each). (E) Schematic of BP pool increases through self-amplification and role of miR-137 in SL neurogenesis and expansion across evolution. Data are represented as mean ± SEM. Biological replicates are distinguished by circles in the bar plots. One-way ANOVA (D). ^∗^p < 0.05, ^∗∗^p < 10^−2^, ^∗∗∗^p < 10^−3^, ^∗∗∗∗^p < 10^−4^.

Focusing on miR-137, we sought to identify cell-type-specific genetic programs that were directly or indirectly regulated by this microRNA by performing single-cell-RNA sequencing 24 h following expression (GEO: GSE159596). A scrambled (scram) version of miR-137 was used in control experiments. We distinguished APs, BPs, newborn (N_0_) neurons, and differentiating (N_1_) neurons by their transcriptional identity (Telley et al., 2019; Figure 2A). As expected, and confirming the role of miR-137 in expansion of the BP pool, BPs were present in higher proportions among progenitors in miR-137 compared with scram conditions (Figure 2B). To identify genes induced and repressed in each cell type, we designed a support vector machine learning approach, which revealed cell type specificity in genes regulated by miR-137 (Figure 2C, left); BPs were most affected, followed by N_0_ neurons and APs (Figure 2C, right). Focusing on BPs, we next analyzed the most strongly (directly and indirectly) repressed and induced genes. Repressed genes were mostly associated with mitochondrial activity and neurogenic processes, whereas induced genes were related to cell cycle progression (Figure S2A). Using gene expression data across corticogenesis (Telley et al., 2019), we found that miR-137 overexpression-induced genes were mainly expressed by progenitors, whereas repressed genes were expressed by neurons (Figure S2B). Accordingly, control neurons showed higher expression of BP repressed genes after miR-137 overexpression than control APs (Figure S2C). These results suggest that miR-137 promotes proliferative and represses neurogenic properties of BPs.

Human BP cells also retain high levels of proliferative genes compared with APs (Figure S2D), suggesting potential evolutionary relevance for the observed effects. This is consistent with the evolutionary profile of miR-137 because 87% of the most induced BP genes and 90% of the most repressed BP genes were expressed in the germinal layer of the ferret during SL neurogenesis (Figures S2F and S2G, respectively). As a proof of principle for the functional relevance of genes directly or indirectly regulated by miR-137 in BPs, we focused on two genes, *Cd63* (among the most induced genes in BPs; Figure S2A) and *Myt1l* (among the most repressed genes in BPs; Figure S2A), which encode an extracellular matrix receptor (Tschoepe et al., 1990) and a transcription factor critical for neuronal identity (Mall et al., 2017), respectively. Consistent with a role in proliferation, gene overexpression led to an increase (by *Cd63*) or decrease (by *Myt1l*) in progenitor numbers and BP/AP ratio (Figures 2D and S2E). Finally, as a proof of principle for direct repression of gene expression by miR-137, we performed a luciferase assay on *Osbpl6* , which has the predicted target sequence for miR-137 in its 3′UTR (miRWalk, Targetscan, and miRDB prediction algorithms), confirming this effect (Figure S2H). These results indicate that miR-137 regulates key genetic programs promoting BP generation and proliferation (Figure 2E).

### miR-137 overexpression promotes SL-type neuron fate

**Figure 3:**
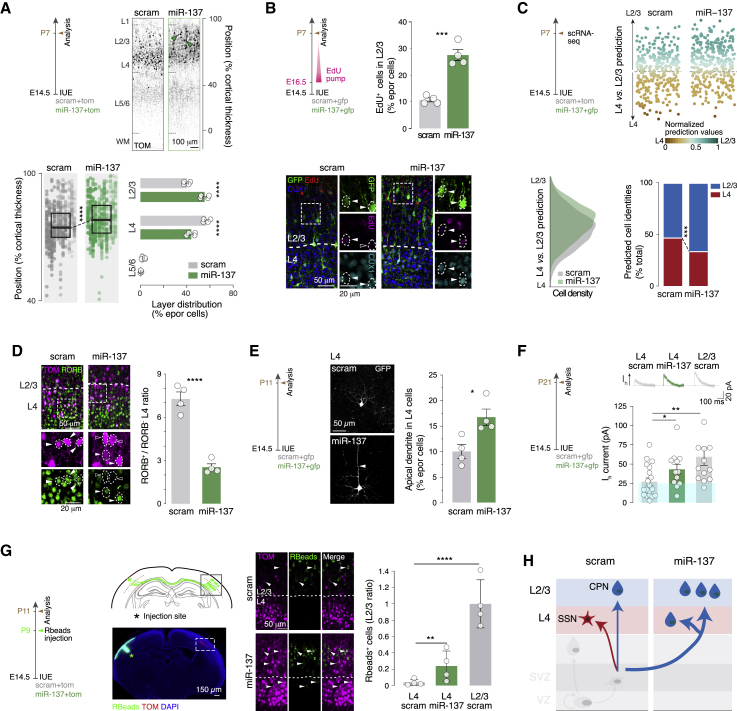
miR-137 promotes expansion of L2/3 by increasing late neurogenesis and reprogramming L4 neurons (A) Radial position of neurons electroporated with scram or miR-137 on P7 (scram and miR-137, n = 4 each). Note the shift of position toward L2/3 under the miR-137 condition. (B) EdU labeling of neurons born after E16.5 upon miR-137 overexpression. Bottom: representative micrographs of EdU staining on P7 in SLs labeled with CUX1 (scram and miR-137, n = 4 each). miR-137 increases late neurogenesis. Electroporated cells positive (filled arrowheads) and negative (empty arrowheads) for EdU are highlighted. (C) scRNA-seq of P7 scram and miR-137 neurons electroporated on E14.5 and of P7 scram neurons electroporated on E14 (L4) and E15.5 (L2/3) (scram and miR-137, n = 3 each). L4 versus L2/3 molecular identities of E14.5 electroporated scram and miR-137 cells were calculated using the scram L4 versus L2/3 prediction model (top). Bottom: prediction model cell density (left) and proportion of cells with L4 versus L2/3 identities (right) under scram and miR-137 conditions. (D) RORB expression in L4 on P7. Left: representative micrographs of immunohistochemistry against RORB. Right: quantification of RORB^+^ versus RORB^−^ cells in L4 electroporated cells (scram and miR-137, n = 4 each). Electroporated cells positive (filled arrowheads) and negative (empty arrowheads) for RORB are highlighted. (E) Morphology of L4 neurons on P11. Left: representative micrographs of L4 scram and miR-137 IUE neurons on P11. Right: quantification of L4 neurons with an apical dendrite (arrowhead) (scram and miR-137, n = 4 each). (F) I_h_ current in P21 L4 and L2/3 scram and L4 miR-137 neurons (L4 scram, n = 19 cells from 3 animals; L4 miR-137, n = 13 cells from 3 animals; L2/3 scram, n = 12 cells from 3 animals). Top right: representative traces. (G) Retrograde labeling of callosally projecting neurons using retrobeads (Rbeads). Left: experimental design. Rbeads were stereotaxically injected into the cortex contralateral to the electroporation site on P9. Right: quantification of Rbead^+^ electroporated cells in L4 and L2/3 under scram and miR-137 conditions (scram and miR-137, n = 4 each). Data were normalized by the number of Rbead^+^ cells under L2/3 scram condition. Arrowheads, Rbead-electroporated cells. (H) Schematic of the role of miR-137 in L2/3 expansion. SSN, spiny stellate neuron; CPN, callosally projecting neuron. Data are represented as mean ± SEM. Biological replicates are distinguished by circles in the bar plots. One-way ANOVA (A, bottom left), two-way ANOVA (A, bottom right; D; and G), Fisher’s chi-square test (C), and unpaired t test (B, E, and F). ^∗^p < 0.05, ^∗∗^p < 10^−2^, ^∗∗∗^p < 10^−3^, ^∗∗∗∗^p < 10^−4^.

We next examined the fate of neurons generated following miR-137 overexpression. As normal corticogenesis unfolds, BP numbers and SVZ size increase so that a significant fraction of SL neurons is born from BPs as L2/3 neurons are being generated (Noctor et al., 2004; Kowalczyk et al., 2009; Taverna et al., 2014). We thus hypothesized that expansion of BP numbers by miR-137 would promote L2/3 neuron fate. To investigate this possibility, we first examined the laminar distribution of neurons born following miR-137 IUE on E14.5, the time of birth of L4 neurons, and analyzed it on P7, when neuronal migration is essentially complete. Although control neurons were predominantly located in L4, neurons in the E14.5 miR-137 overexpression condition were located more superficially, including within L2/3 (Figure 3A). We next examined whether the increase in L2/3 neuron numbers reflected an increase in late neurogenesis and/or a shift from L4 to L2/3 identity. We first chronically applied 5-ethynyl-2′-deoxyuridine (EdU) to birth label neurons as BPs are dividing (E16.5; Figure 3B). The fraction of neurons labeled in the miR-137 overexpression condition was 3 times higher than in the control condition (30% versus 10%), suggesting that miR-137 amplifies neuronal production (Figure 3B). To address the molecular L4 versus L2/3 identity on P7 upon miR-137 overexpression, we performed single-cell RNA sequencing (scRNA-seq) of P7 neurons after E14.5 electroporation of scram or miR-137 (Figure 3C; GEO: GSE159596). We used a control dataset of P7 neurons electroporated with scram on E14.0 (putative L4 neurons) and E15.5 (putative L2/3 neurons) to predict the molecular identities of miR-137-overexpressing neurons on P7 using a machine learning classifier (Figures 3C and S3A). This approach showed an increased proportion of neurons with L2/3-type identity in the miR-137 overexpression condition (Figure 3C). Supporting this finding, L4-located neurons under the E14.5 miR-137 condition did not express the L4 neuron marker RORB (Figures 3D, S3B, and S3C) and instead expressed high levels of the L2/3 neuron-enriched protein BRN2 (Figure S3D). Consistent with a functional relevance of this L4-to-L2/3 shift in molecular programs, L4 neurons in the E14.5 miR-137 overexpression condition often displayed an apical dendrite (which is typically absent in L4 neurons and is present in L2/3 neurons; Figure 3E) and displayed a hyperpolarizing-activated current (I_h_ current), an electrophysiological feature normally not found in L4 neurons and instead present in L2/3 neurons (Figure 3F; (Klingler et al., 2019) De la Rossa et al., 2013). Retrograde labeling from the contralateral hemisphere revealed the presence of interhemispheric projections in L4-located neurons following miR-137-overexpression on E14.5, a typical feature of L2/3-type neurons (Figure 3G). As a proof-of-principle demonstration of direct repression of gene expression by miR-137, we performed a luciferase assay on *Cadm2*, which has the predicted target sequence for miR-137 in its 3′UTR (miRWalk, Targetscan, and miRDB prediction algorithms), confirming this effect (Figure S3E). These results indicate that amplification of the BP pool by miR-137 results in expansion of neurons with laminar, molecular, anatomical, and functional features of L2/3-type neurons (Figure 3H).

### miR-122 regulates unfolding of postmitotic neuronal differentiation programs

**Figure 4:**
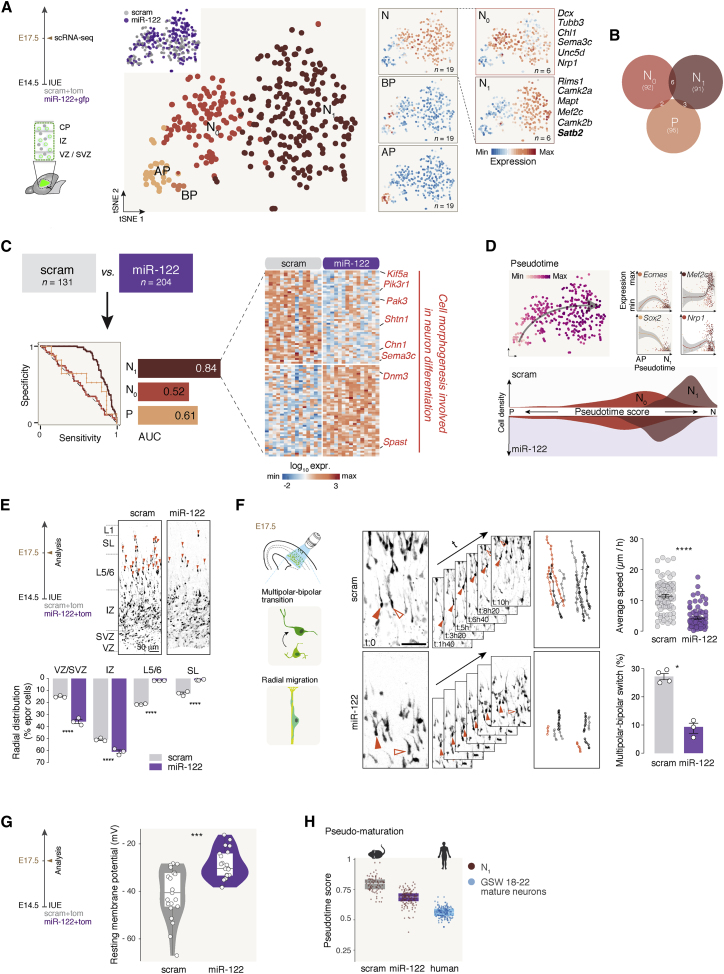
miR-122 regulates unfolding of postmitotic neuronal differentiation programs (A) t-SNE representation of E17.5 scram and miR-122 E14.5 IUE scRNA-seq data reveals transcriptional organization of the cells according to their differentiation status. APs, BPs, N_0_ neurons, and differentiating neurons (N_1_) can be distinguished by their combinatorial expression of key marker genes (scram and miR-122, n = 3 each). (B) Machine learning approach to identify cell-type-specific core sets of genes classifying neurons and progenitors under miR-122 and scram conditions. Shown are shared differentially expressed genes between cell types. (C) Left: machine learning prediction score for each cell type, shown as AUC. Note the highest score in N_1_ neurons. Right: heatmap of the top miR-122-induced and -repressed genes in N_1_ neurons. (D) Pseudotime analysis of scram and miR-122 single cells. Top left: pseudotime values of single cells shown in the t-SNE space. Top right: marker genes of APs, BPs, N_0_ neurons, and N_1_ neurons expressed along pseudotime. Bottom: density plot of pseudotime values of N_0_ and N_1_ neurons under scram and miR-122 conditions. (E) Radial position of SL neurons at E17.5 after scram and miR-122 IUE (scram and miR-122, n = 3 each). Orange arrowheads indicate neurons in cortical plate (CP). (F) Live imaging of scram and miR-122 IUE neurons on E17.5. Left: experimental design. Center: images illustrating neuron movement tracking under scram and miR-122 conditions. Right: quantification of the average speed of migration and the multipolar-bipolar transition (MBT) (scram, n = 4; miR-122, n = 3). (G) Resting membrane potential of scram and miR-122 migrating neurons on E17.5 (scram, n = 20 cells from 3 animals; miR-122, n = 21 cells from 3 animals). (H) Pseudotime value predictions of human SL differentiating neurons from gestational week (GSW) 18–22 fetuses using the mouse model. Data are represented as mean ± SEM. Biological replicates are distinguished by circles in the bar plots. Two-way ANOVA (E), Kruskal-Wallis test (F), and unpaired t test (G). ^∗^p < 0.05, ^∗∗^p < 10^−2^, ^∗∗∗^p < 10^−3^, ^∗∗∗∗^p < 10^−4^. Human scRNA-seq data are from Nowakowski et al. (2017).

As indicated above, miR-122 does not affect progenitor proliferation (Figures 1B, 1C, and S1F) but can still act on differentiation of N_0_ neurons in the SVZ during late neurogenesis. To investigate a potential postmitotic effect of miR-122, we overexpressed this transcript using IUE, as described above. Using the same approach as for miR-137, we performed scRNA-seq 72 h following miR-122 expression (GEO: GSE159596), distinguishing APs, BPs, N_0_ neurons, and N_1_ neurons based on their transcriptional identity (Figure 4A). The support vector machine learning approach identified induced and repressed cell-type-specific pools of genes directly and indirectly regulated by miR-122 overexpression (Figure 4B). Consistent with a postmitotic effect, N_1_ neurons were the most affected by miR-122 (Figure 4C). In miR-122 overexpression, regulated genes within these neurons had ontologies relating to cell morphogenesis and differentiation, including microtubule cytoskeleton structure and axon development (Figures 4C and S4A). Consistent with a role in neuronal maturation, pseudotime analysis revealed that miR-122-overexpressing neurons were transcriptionally less mature than their control counterparts, suggesting that miR-122 overexpression delays neuronal maturation (Figure 4D). To assess the functional correlates of these transcriptional changes, we analyzed key neuronal postmitotic features such as positioning, migration dynamics, and expression of mature molecular programs. This first revealed that miR-122-overexpressing neurons were mostly found between the SVZ and the lower intermediate zone (IZ), whereas only few cells reached the cortical plate (Figure 4E). In live imaging of acute cortical slices, migration of miR-122-overexpressing neurons was delayed compared with that of control neurons (Figures 4F and S4B). Consistent with a slower maturation process, miR-122 N_0_ neurons also failed to hyperpolarize their membrane potential (Figure 4G; Picken Bahrey and Moody, 2003). Further supporting slower differentiation, SATB2 (a marker of mature SL neurons) immunofluorescence levels were lower in miR-122-overexpressing neurons than in controls (Figure S4C). We then compared mouse and human embryonic neurons during SL neurogenesis (Nowakowski et al., 2018; gestational week [GSW] 18–22 mature neurons, which we compared with N_1_ cells). Projection of human neurons in the mouse pseudo-maturation axis showed that they were even more immature than miR-122-overexpressing neurons (Figures 4H and S4D), consistent with longer differentiation times in humans (Linaro et al., 2019). The effect of miR-122 on genes that control neuronal maturation was further examined by comparing the dynamics of the repressed genes in the N_1_ population in mice and humans (Figure S4E); the vast majority of the genes repressed by miR-122 overexpression were upregulated during normal neuronal development (93% in mice and 88% in humans; Figure S4E). The evolutionary relevance of miR-122 was further strengthened by comparison with a ferret database (De Juan Romero et al., 2015), where 92% of the most induced genes in N_1s_ and 80% of the most repressed genes in N_1s_ were expressed in ferret during SL neurogenesis (Figures S4F and S4G respectively). Finally, to identify a direct target of miR-122 in N_1_ neurons, we performed a luciferase assay on *Zfp68*, which has the predicted target sequence for miR-122 in its 3′UTR (miRWalk, Targetscan, and miRDB prediction algorithms), confirming this effect (Figure S4H). These results suggest an evolutionarily conserved postmitotic role for miR-122 in slowing the pace of neuronal differentiation.

### miR-122 overexpression promotes L2/3-type neuron fate

**Figure 5:**
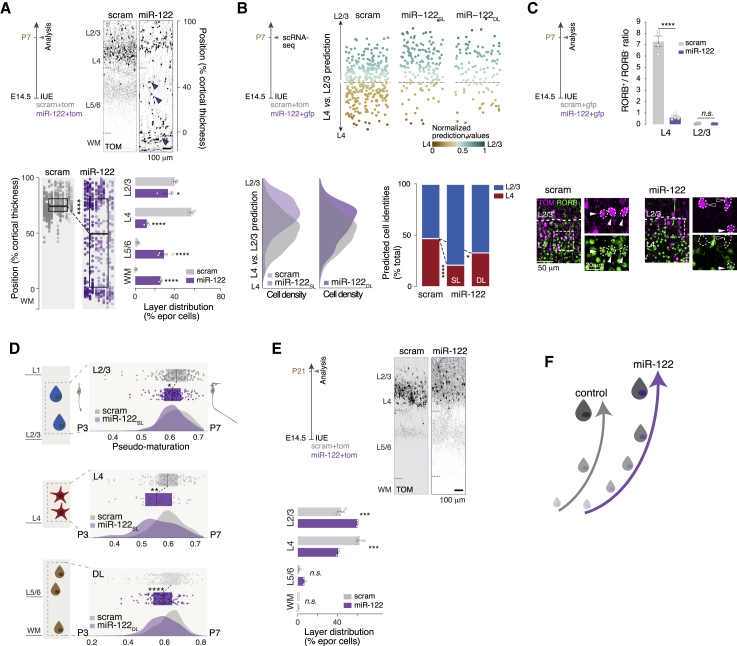
miR-122 overexpression slows the pace of neuronal differentiation and promotes L2/3-type neuron fate (A) Radial position of P7 neurons electroporated with scram or miR-122 at E14.5 (scram and miR-122, n = 4). Arrowheads, neurons in DLs or white matter (WM). (B) scRNA-seq of P7 scram and miR-122 neurons electroporated on E14.5 and of P7 scram neurons electroporated at E14 (L4) and E15.5 (L2/3) (scram and miR-122, n = 3). L4 versus L2/3 molecular identities of E14.5 electroporated scram and miR-122 cells were calculated using the scram L4 versus L2/3 prediction model (top). Bottom: prediction model cell density (left) and proportion of cells with L4 versus L2/3 identities (right) in scram and miR-122 conditions. miR-122 cells predicted as positioned in superficial layers (SLs) or deep layers (DLs) are represented separately. (C) RORB expression in L4 on P7. Top: quantification of RORB^+^ versus RORB^−^ cells in L4 electroporated cells (scram and miR-122, n = 4). Bottom: representative micrographs of immunohistochemistry against RORB. Electroporated cells positive (filled arrowheads) and negative (empty arrowheads) for RORB are highlighted. (D) Pseudo-maturation score calculated using a model of P3 versus P7 L4 and L2/3 neurons (electroporated with scram on E14 and E15.5, respectively). L2/3 and L4 neurons are represented separately. miR-122 neurons located in DLs were compared with all scram SL neurons (light gray). (E) Radial position of P21 neurons electroporated with scram or miR-122 at E14.5 (scram and miR-122, n = 3). Note the absence of neurons in DLs in the miR-122 condition. (F) Schematic of the role of miR-122 in slowing down SL neuron maturation. Data are represented as mean ± SEM. Biological replicates are distinguished by circles in the bar plots (A, C, and E). One-way ANOVA (A, bottom left), two-way ANOVA (A, bottom right; C; and E), and Fisher’s chi-square test (C). ^∗^p < 0.05, ^∗∗^p < 10^−2^, ^∗∗∗^p < 10^−3^, ^∗∗∗∗^p < 10^−4^.

We next examined the fate of neurons generated following miR-122 overexpression. First, we examined the laminar distribution of neurons at P7 following miR-122 IUE on E14.5. In contrast to the control condition, upon miR-122 overexpression, half of the neurons were mispositioned in deep layers (DLs) and white matter (WM), whereas the other half settled correctly in the SL (Figure 5A). Although L2/3 neuronal distribution was similar to that of controls, L4 was essentially depleted of neurons under the miR-122 condition, suggesting that the majority of misplaced neurons in the DL/WM were prospective L4 neurons. Chronic EdU labeling from E15.5 (when L2/3 neurons are born) showed that very few EdU^+^ miR-122-overexpressing cells were found in the DL and WM on P3 (when migration of SL neurons is largely complete), supporting this possibility (Figure S5A). We next investigated the molecular identity of miR-122-overexpressing neurons (Figure S5; STAR Methods; GEO: GSE159596), which revealed that miR-122 neurons in the SL and DL displayed an increased ratio of L2/3-predicted to L4-predicted cells (Figure 5B), suggesting that slowing down L4 neuron differentiation by miR-122 (Figure 4) results in acquisition of an L2/3 neuron-like molecular identity. Supporting this finding, L4-located miR-122-overexpressing neurons on P7 did not express the L4 neuron marker RORB (Figure 5C). We next wanted to determine whether miR-122 SL and DL neurons still showed immaturity traits on P7. We used a postnatal maturation model where neurons of L2/3 (E15.5 born) and L4 (E14.0 born) were collected on P3 and P7 (Figure S5C; GEO: GSE159596). Analysis of this dataset revealed that miR-122 neurons had a lower pseudo-maturation score than control neurons on P7, whatever their laminar position, with DL neurons being most immature (Figure 5D). In contrast to results obtained with miR-137 (Figure S3D), L4-located miR-122-overexpressing neurons failed to increase BRN2 expression, perhaps reflecting delayed maturation (Figure S5F). Furthermore, miR-122-overexpressing neurons showed a significant reduction in CUX1 expression, consistent with CUX1 being a known direct target of miR-122 (Xu et al., 2010; Figure S5G). These results suggest that mispositioned miR-122 neurons may still be migrating to their final position and therefore express more immature transcriptional programs. On P21, few miR-122 neurons remained in the DL or WM, and their distribution was shifted toward L2/3 compared with scram neurons in the SL (Figures 5E and S5D). Because we did not observe increased cell death in miR-122 neurons (Figure S5E), and in line with the shift in neuronal identity reported above, these results suggest protracted migration upon miR-122 overexpression and preferential settling in L2/3. These results reveal that miR-122 affects the maturation pace of SL neurons and suggest that slowing genetic programs by miR-122-overexpressing L4-type neurons is associated with acquisition of an L2/3-type identity (Figure 5F).

## Discussion

Our results reveal that miR-137 and miR-122 expression in mice affects cortical development by acting on pre- and postmitotic cells, respectively. miR-137 mostly acts on BPs, maintaining their proliferative state, including through indirect upregulation of the extracellular matrix receptor *Cd63* and repression of *Myt1l*, a transcription factor promoting neuronal identity. On the other hand, miR-122 slows down neuronal maturation.

We identify miR-137 and miR-122 as being expressed in the ferret oSVZ during SL neurogenesis. In the oSVZ, BPs highly proliferate to sustain cortical expansion and folding (Pilz et al., 2013; Dehay et al., 2015). Recent studies have shown that both BP types (bRG and IP) have increased proliferation capacity (Kalebic et al., 2019; Betizeau et al., 2013), mainly through interactions with the extracellular matrix (Arai et al., 2011; Fietz et al., 2012).

Here we show that miR-137 maintains BPs in a proliferative state not only through maintenance of cell- cycle genes and repression of neuronal genes but also through indirect induction of an extracellular matrix receptor mediating the response to the integrin signaling pathway (*Cd63*), which is known to promote proliferation of BPs (e.g., the integrin avb3 and b1 pathways; Fietz et al., 2010; Kalebic et al., 2019; Stenzel et al., 2014).

Progenitor division modes change as corticogenesis proceeds. L2/3 neuron generation mostly relies on BPs, whereas a significant proportion of DL neurons are born directly from APs. Overexpression of cell cycle genes, such as cyclins, in the mouse embryonic cortex can amplify the number of BPs and increase generation of L2/3 neurons (Lange et al., 2009; Pilaz et al., 2009). Consistent with these findings, we show that miR-137 overexpression triggers BP proliferation at the peak of BP-derived neurogenesis (i.e., during L2/3 neurogenesis) and at earlier time points (i.e., during DL neurogenesis). This supports an evolutionary role of this miRNA in the disproportionate increase in L2/3 neuron numbers in gyrencephalic species.

The protracted duration of corticogenesis in more evolved species is reflected by a longer progenitor cell- cycle duration (Bystron et al., 2006; Caviness et al., 1995; Rakic, 2009; Lukaszewicz et al., 2005; Betizeau et al., 2013) and a longer neurogenic period (Lewitus et al., 2014; Wilsch-Bräuninger et al., 2016). In addition, postmitotic neuronal maturation is longer in more evolved species, and human neurons transplanted into the mouse cortex retain a slow developmental pace (Linaro et al., 2019). In our study, we show that miR-122 overexpression acts postmitotically by modifying the transcriptional profile of N_0_ neurons during SL neurogenesis, along with a slower differentiation and migration pace and a shift in molecular identity from L4 to L2/3. In evolutionary terms, lengthened maturation is associated with protracted differentiation of synapses and dendrites, increased synaptic plasticity, circuit integration, and specialization, which could underlie the extended postnatal plasticity of neocortical circuits (Petanjek et al., 2011; Gould, 1992). It would be interesting in further studies to characterize in more detail the postmitotic mechanisms that underlie the slower differentiation and elongated neuronal maturation triggered by miR-122 overexpression.

Non-coding RNAs have emerged over the last decade as a critical source of genetic regulation across species (Fernández et al., 2016; Florio et al., 2017). The proportion of non-coding RNAs per genome size increases across evolution (Liu et al., 2013; Taft and Mattick, 2003; Taft et al., 2007), as do numbers of miRNAs and the length of their targeting region (Meunier et al., 2013). The regulatory importance of miRNAs is particularly well studied during neocortical development, and several studies have shown that absence of miRNAs during cortical development leads to severe malformations (Choi et al., 2008; Cuellar et al., 2008; Damiani et al., 2008; Davis et al., 2008; De Pietri Tonelli et al., 2008; Kim et al., 2007; Stark et al., 2008). Gyrencephalic species have an enriched array of miRNAs (Johnson et al., 2015; Moreau et al., 2013), in particular across the different germinal layers (Arcila et al., 2014). Here we found that miR-137 and miR-122 overexpression modifies the transcriptional landscape of mouse BPs and N_0_ neurons and induces features associated with increased neuronal production in gyrencephalic species. A shared role of miR-137 and miR-122 in mice (following overexpression) and gyrencephalic species is supported by essentially shared target genes and Gene Ontology families between mouse and human (Figure S1C and S1D), suggesting that levels of expression, rather than fundamental differences in their target genes, underlie this neurogenic effect.

miR-137 acts during SL neurogenesis, whereas miR-122 is involved in SL neuronal differentiation, triggering molecular cascades in gyrencephalic species with expansion of L2/3 identity. It will be interesting in future studies to investigate the mechanisms through which changes in the pace of molecular differentiation programs lead to distinct cellular fates.

### Limitations of the study

microRNAs regulate mRNA translation. Here we used scRNA-seq experiments to address the cortical cell identity, but effects at the protein level remain largely unexamined. Direct regulation of specific target genes by miRNAs could also be addressed using luciferase assays.

miR-137 and miR-122 are expressed at very low levels in mice. To address how downregulation of those miRNAs affects corticogenesis, loss-of-function experiments using gyrencephalic species, such as the ferret, or in human cortical organoids, could be performed to obtain further insights into its relevance in cortical evolution.

## STAR★Methods

### Key resources table

**Table:** 

REAGENT or RESOURCE	SOURCE	IDENTIFIER
**Antibodies**		

Chicken anti-GFP	Abcam	Cat.N: #AB13970; RRID:AB_300798
Goat anti-TOM	Sicgen	Cat.N: #AB8181-200; RRID:AB_2722750
Rabbit anti-RFP	Abcam	Cat.N: #AB62341; RRID:AB_945213
Mouse anti-PAX6	Thermofischer Scientific	Cat.N: #MA1-109; RRID:AB_2536820
Rat anti-EOMES	Invitrogen	Cat.N: #14-4875-82; RRID:AB_11042577
Rat anti-EOMES	eBioscience	Cat.N: #144875-82; RRID:AB_11042577
Rabbit anti-KI67	Abcam	Cat.N: #AB15580; RRID:AB_805388
Rabbit anti-NEUROD2	Abcam	Cat.N: #AB104430; RRID:AB_10975628
Mouse anti-RORB	Perseus Proteomics	Cat.N: #PP-N7927-00; RRID:AB_1964364
Rabbit anti-CUX1	Santa Cruz	Cat.N: #sc13024; RRID:AB_2261231
Rabbit anti-CASPASE3	Cell signaling technology	Cat.N: #9662S; RRID:AB_331439
mouse anti-SATB2	Abcam	Cat.N: #AB51502; RRID:AB_882455

**Biological samples**		

Mouse CD1 embryonic and postnatal brains	This paper	N/A
Ferret postnatal brains	This paper	N/A
Healthy P1 ferret brain tissue	Animal Facilities, Universidad Miguel Hernández	N/A

**Chemicals, peptides, and recombinant proteins**		
endotoxin-free Maxiprep kit	QIAGEN	Cat.N: #12362
5-Bromo-20-deoxyuridine (BrdU)	Sigma	Cat.N: #B5002
5-Ethynyl-2′-deoxyuridine (EdU)	Sigma	Cat.N: # 900584
red Retrobeads! IX	Lumafluor	N/A
Hoechst	Thermofischer Scientific	Cat.N: #H1399
Pronase	Sigma	Cat.N: # P5147
70 mm cell strainer	Clearline	Cat.N: #141379C
Draq7TM	Beckerman Coulter	Cat.N: #B25595
RNeasy kit	QIAGEN	Cat.N: #74034
SMARTseq v4 kit	Clontech	Cat.N: #634888
pmirGLO Dual-Luciferase miRNA target expression vector	Promega	Cat.N: E1330
Dual-Glo Luciferase Assay System	Promega	Cat.N: E2920
Polyethylenimine	Polysciences	Cat.N:24765-1

**Critical commercial assays**		

Osmotic pump	Alzet	Cat.N: 1003D
Alkaline phosphatase coupled anti-digoxigenin Fab fragments	Roche	Cat.N: 11093274910

**Deposited data**		

E30-34-P2 ferret microarray	Martinez-Martinez et al., 2016	GEO: GSE63203
P2 ferret microarray	De Juan Romero et al., 2015	GEO: GSE60687
ScRNA-Seq E14.5-15.5, E14.5-17.5, E14.5-P7, E14.0-P7, E15.5-P7, E14.0-P3, E15.5-P3	GEO	GEO: GSE159596

**Experimental models: Cell lines**		

HEK293T cells	Merck	Cat#12022001

**Experimental models: Organisms/strains**		

CD1	Charles River	N/A
Ferret	Marshall Bioresources	N/A

**Oligonucleotides**		

Primers for Cadm2 3’UTR: forward: ACGAGCTCGCTAGCCTCGAGTCTAGAAG TGGCACCAAGTACACAC; reverse: ATCAGCTTGCATGCCTGCAGGTCGACGGA GAAAAGCGAGGAGGAG	IDT	N/A
Primers for Osbpl6 3’UTR: forward: ACGAGCTCGCT AGCCTCGAGTCTAGGAATGAAGTCCAGGGGGTGG; reverse: ATCAGCTTGCATGCCTGCAGG TCGAGAGTGGGTTTCGGGAAGCAT	IDT	N/A
Primers for Zfp68 3’UTR: forward: ACGAGCTCGC TAGCCTCGAGTCTAGTAAGGGGCAGCATGAGAAGC; reverse: ATCAGCTTGCATGCCTGCAGGTCGA ACAGGGTCCCCTTAGCTGTA	IDT	N/A
Primers for pUG018: forward: TCGAGGAG AATCCTGGCCCAGATCTTATGGACGTGGAC TCTGAGGAG; reverse: TGGCAGAGGGAAAAAG ATCTAAGCATGAGTCACCACTAGAGC	IDT	N/A
Primers for pUG016: forward: TCGAGGAG AATCCTGGCCCAGATCTTATGGCGGTGGAA GGAGGAATG; reverse: TGGCAGAGGGAAAAAG ATCTCTACATTACTTCATAGCCACTTCG	IDT	N/A
Primers for DFRS122: forward: TAACAAACA CCATTGTCACACTCCAGGCGCGCCCCAAAC ACCATTGTCACACTCCAGGCCGG; reverse: TAATTGTTTGTGGTAACAGTGTGAGGTCCGCGCGG GGTTTGTGGTAACAGTGTGAGGTCC	IDT	N/A
Primers for DFRS137: forward: TAACTACG CGTATTCTTAAGCAATAAGGCGCGCCCTA CGCGTATTCTTAAGCAATAAGGCCGG; reverse: TAATTGATGCGCATAAGAATTCGTTA TTCCGCGCGGGATGCGCATAAGAATTC GTTATTCC	IDT	N/A
Primers for pSilencer-U6-miR-122: forward: GATCCGCAAACACCTTTGTCAGTCTGCAT TCAAGAGATGGAGTGTGACAATGGTGTT TGTTTTTTGGAAA; reverse: AGCTTTTC CAAAAAACAAACACCATTGTCACACT CCATCTCTTGAATGCAGACTGACAAA GGTGTTTGCG	IDT	N/A
Primers for pSilencer-U6-miR-137: forward: GATCC CCTACGTGTATTCTTAACGAACAATTCAAGAGATTA TTGCTTAAGAATACGCGTAGTTTTTTGGAAA; reverse: AGCTTTTCCAAAAAACTACGCGTATTCTTAAGC AATAATCTCTTGAATTGTTCGTTAAGAATAC ACGTAGGG	IDT	N/A
HSA-MIR-122 miRCURY™ LNA™ microRNA ISH Detection Probe: /5DiGN/AAACACCATTGTCAC ACTCCA/3DiG_N/	Qiagen	#339111-YD00619864-BCG
HSA-MIR-137 miRCURY™ LNA™ microRNA ISH Detection Probe: /5DiGN/ACGCGTATTCTTAAGC AATA/3DiG_N/	Qiagen	#339111-YD00610858-BCG

**Recombinant DNA**		

pSilencer-U6-miR-137	This paper	N/A
pSilencer-U6-miR-122	This paper	N/A
dUP-Cd63	This paper	N/A
dUP-Myt1l	This paper	N/A
DFRS-137	This paper	N/A
DFRS-122	This paper	N/A
pmirGLO + Cadm2 3’UTR	This paper	N/A
pmirGLO + Osbpl6 3’UTR	This paper	N/A
pmirGLO + Zpf68 3’UTR	This paper	N/A

**Software and algorithms**		

R	N/A	https://www.r-project.org/
GraphPad Prism	GraphPad software	https://www.graphpad.com
ImageJ	NIH	https://imagej.nih.gov/ij/
Adobe Photoshop	Adobe	https://www.adobe.com/products/photoshop.html
Adobe Illustrator	Adobe	https://www.adobe.com/products/illustrator.html

### Resource availability

#### Lead contact

Alexandre Dayer passed away during the submission process of this work. Further information and requests for resources should be directed to and fulfilled by his representative, Denis Jabaudon (denis.jabaudon@unige.ch).

#### Materials availability

This study generate DNA expression vectors unique material. For further inquiries, please address to the corresponding author or to the lead contact.

### Experimental model and subject details

#### Mouse

All experimental procedures were approved by the Geneva Cantonal Veterinary Authority and performed according to the Swiss law. Embryonic day (E) 0.5 was established as the day of vaginal plug. Wild-type CD1 mice were provided by Charles River Laboratories. Male and female embryos between E12.5 and E15.5 were used for the *in utero* electroporations, and pups between postnatal day (P) 0 and P21 for the postnatal experiments. Pregnant dams were kept in single cages and pups were kept with their mothers until P21, in the institutional animal facility under standard 12:12 h light / dark cycles.

#### Ferret

The miR expression data was obtained from De Juan Romero et al., 2015 and Martinez-Martinez et al., 2016 and is available on GEO at https://www.ncbi.nlm.nih.gov/geo/query/acc.cgi?acc=GSE60687 and https://www.ncbi.nlm.nih.gov/geo/query/acc.cgi?acc=GSE63203.

#### Cell lines

HEK-T293 cells were maintained in culture flasks (Falcon) with Dulbecco's Modified Eagle Medium (DMEM, GIBCO) supplemented with 1% penicillin-streptomycin and 10% fetal calf serum (FCS, GIBCO) in an incubator (37°C, 5% CO2).

### Method details

#### In *utero* electroporation

Timed pregnant CD1 mice were anaesthetized with isoflurane (5% induction, 2.5% during the surgery) and treated with the analgesic Temgesic (Reckitt Benckiser, Switzerland). Embryos were injected unilaterally with 700 nL of DNA plasmid solution (diluted in endofree PBS buffer and 0.002% Fast Green FCF (Sigma)) into the lateral ventricle. Embryos were then electroporated by holding each head between circular tweezer-electrodes (5 mm diameter, Sonidel Limited, UK) across the uterine wall, while 5 electric pulses (35 V for E12.5, 40 V for E13.5, 45 V for E14.5, 50 V for E15.5, 55 V for E17.5 and E18.5, 50 ms at 1 Hz) were delivered with a square-wave electroporator (Nepa Gene, Sonidel Limited, UK).

#### Plasmids

Injected plasmids were: pUB6-GFP and pUB6-TOM (0,5 mg/mL); pSilencer-U6-scram, pSilencer-U6-miR-137 and pSilencer-U6-miR-122 (1 mg/mL); dUP-Cd63 and dUP-Myt1l (2 mg/mL, subcloned from double UP mClover to Scarlet, Addgene #125134, (Taylor et al., 2020)); DFRS, DFRS-137 and DFRS-122 (1 mg/mL, subcloned from DFRS, De Pietri Tonelli et al., 2006), pmiRGLO and pmirGLO + Cadm2 3′UTR and pmirGLO + Osbpl6 3′UTR and pmirGLO + Zfp68 3′UTR (40 ng/well).

A vector backbone pSilencer 2.1 was used to clone the pSilencer-U6-miR-137 and the pSilencer-U6-miR-122. MiR-137 and miR-122 sequence is flanked by BamHI and HindIII excision sites, allowing the insertion into the pSilencer 2.1-U6 *neo* Vector (already linearized; Ambion) using the In-Fusion Kit (Clontech). The mir-137 and miR-122 sequence was thus under the control of the constitutive human RNA Polymerase III promoter U6.

The Dual-Fluorescent-GFP-Reporter/mRFP-Sensor plasmid (DFRS) is able to detect strong and weak expressions of miRNAs at a single cell level (De Pietri Tonelli et al., 2006). With The low endogenous level of miR-137 and miR-122 can be detected, as well as the effect of the gain of function, which allows the validation of the pSilencer-U6-miR-137 and the pSilencer-U6-miR-122 plasmids. In DFRS, both the red fluorescent protein (RFP) gene and the green fluorescentprotein (GFP) gene were driven by the identical simian vacuolating virus 40 (SV40) promoter, which induced a constitutive expression of the genes under this promoter (Oellig and Seliger, 1990). The GFP was the reporter, and thus was not affected by any miRNA. The 3′UTR of RFP gene contained the control cassette (unaffected by any miRNA), or the miR-137 or miR-122 target cassette (sensible to the expression of miR-137 or miR-122). The RFP transcript was thus the sensor, its translation being affected (DFRS137 or DFRS122) or not (DFRS control) by the presence of miR-137 or miR-122 in the cell. The DFRS control plasmid was constructed from the pGEM shuttle plasmid, which contained a control cassette (Unc-54 3′UTR, C. elegans), that did not include any miRNA active 3′UTR. Thus, this control cassette was not targeted by any miRNA from the mouse genome. This cassette was amplified (pGEM 3′UTR cassette primers) then removed from the pGEM shuttle plasmid using EcoRI and NotI enzymes and annealed into the DFRS empty plasmid pre-amplified (DFRS empty plasmid primers) and digested by the same enzymes (In-Fusion Kit; Clontech). The control cassette was thus placed on the 3′UTR of the RFP gene.

The DFRS137 and DFRS122 were the sensors of the level of miR-137 and miR-122 in the cell. For this, the 3′UTR of the RFP gene in the DFRS plasmid contained two perfect complementary sequence of miR-137 or miR-122 in tandem. Thus, these sequences were recognized and targeted by miR-137 or miR-122, which induced the degradation of the messenger RNA of the RFP. First, the primers for the complementary sequence of miR-137 or miR-122 were designed (miR-137 or miR-122 target cassette primers), flanked by PacI and FseI restriction sites. The cassette was then inserted by digestion and ligation into the pGEM shuttle plasmid. The complete cassette containing the 3′UTR information was amplified (pGEM 3′UTR cassette primers), digested by EcoRI and NotI and ligated to the DFRS empty plasmid, pre-amplified (DFRS empty plasmid primers) and pre-digested by the same enzymes (In Fusion Kit; Clontech). The miR-137 or miR-122 complementary sequences were thereby located on the 3′UTR of the RFP gene.

A vector backbone (pUG001) was constructed to allow for temporal control of gene expression (*CAG-loxP-mClover3-polyA-loxP-mScarlet-MCS-polyA*). Upon plasmid delivery, only mClover3 is constitutively expressed. As this gene is flanked by *loxP* sites, subsequent delivery of Cre protein results in the excision of mClover3 sequence, allowing for expression of mScarlet and the gene of interest (cloned at the MCS). A vector backbone (Double UP, Addgene #125134, Taylor et al., 2020) was used to allow for temporal control of gene expression (*CAG-loxP-mClover3-polyA-loxP-mScarlet-MCS-polyA*). Genes of interest were PCR amplified from a cDNA library from mouse embryonic brain RNA extracted at E14.5.

Double UP plasmid was modified to permit constitutive expression of the gene of interest, and its ablation upon Cre activation, by including a T2A site downstream of mClover3. The modified plasmid (pUG015) was digested with BglII (ThermoFisher, FD0083) and Gibson Assembly (NEB, E2611S) was used to introduce Cd63 (pUG016) and Myt1L (pUG018) downstream of T2A site.

**Table:** 

Primer_ID	Plasmid	Sequence
Fwd miR-137	pSilencer-U6-miR-137	GATCCCCTACGTGTATTCTTAACGAACAATTCAAGAGATTATTGCT TAAGAATACGCGTAGTTTTTTGGAAA
Rev miR-137		AGCTTTTCCAAAAAACTACGCGTATTCTTAAGCAATAATCTCTTGAA TTGTTCGTTAAGAATACACGTAGGG
Fwd miR-122	pSilencer-U6-miR-122	GATCCGCAAACACCTTTGTCAGTCTGCATTCAAGAGATGGAGT GTGACAATGGTGTTTGTTTTTTGGAAA
Rev miR-122		AGCTTTTCCAAAAAACAAACACCATTGTCACAC TCCATCTCTTGAATGCAGACTGACAAAGGTGTTTGCG
Fwd pGEM 3′UTR cassette	DFRS	ATGGCCGCGGGATTATTAGC
Rev pGEM 3′UTR cassette		ATATGGTCGACCTGCAGGC
Fwd DFRS empty plasmid	DFRS	ATGCTCATCGTGAAAGCGG
Rev DFRS empty plasmid		AAGTCTAGACAGAATTCTAGGCGC
Fwd miR-137 target cassette	DFRS137	TAACTACGCGTATTCTTAAGCAATAAGGCGCGC CCTACGCGTATTCTTAAGCAATAAGGCCGG
Rev miR-137 target cassette		TAATTGATGCGCATAAGAATTCGTTATTCCGCGCG GGATGCGCATAAGAATTCGTTATTCC
Fwd miR-122 target cassette	DFRS122	TAACAAACACCATTGTCACACTCCAGGCGCGCCCC AAACACCATTGTCACACTCCAGGCCGG
Rev miR-122 target cassette		TAATTGTTTGTGGTAACAGTGTGAGGTCCGCGCGGGG TTTGTGGTAACAGTGTGAGGTCC
Fwd_Cd63	pUG016	TCGAGGAGAATCCTGGCCCAGATCTTATGGCGGTGGAAGGAGGAATG
Rev_Cd63		TGGCAGAGGGAAAAAGATCTCTACATTACTTCATAGCCACTTCG
Fwd_Myt1l	pUG018	TCGAGGAGAATCCTGGCCCAGATCTTATGGACGTGGACTCTGAGGAG
Rev_Myt1l		TGGCAGAGGGAAAAAGATCTAAGCATGAGTCACCACTAGAGC
Fwd_Cadm2 3′UTR	pmirGLO + Cadm2 3′UTR	ACGAGCTCGCTAGCCTCGAGTCTAGAAGTGGCACCAAGTACACAC
Rev_Cadm2 3′UTR		ATCAGCTTGCATGCCTGCAGGTCGACGGAGAAAAGCGAGGAGGAG
Fwd_Osbpl6 3′UTR	pmirGLO + Osbpl6 3′UTR	ACGAGCTCGCTAGCCTCGAGTCTAGGAATGAAGTCCAGGGGGTGG
Rev_Osbpl6 3′UTR		ATCAGCTTGCATGCCTGCAGGTCGAGAGTGGGTTTCGGGAAGCAT
Fwd_Zfp68 3′UTR	pmirGLO + Zfp68 3′UTR	ACGAGCTCGCTAGCCTCGAGTCTAGTAAGGGGCAGCATGAGAAGC
Rev_Zfp68 3′UTR		ATCAGCTTGCATGCCTGCAGGTCGAACAGGGTCCCCTTAGCTGTA

#### Injections and continuous drugs administration (EdU)

For chronic administration, an osmotic pump (Alzet, #1003D) was filled with 10 mg/mL solution of EdU (Sigma, #900584) was prepared in 1:1, DMSO: water and placed in the peritoneal cavity at the end of the surgery or at given gestational day (Telley et al., 2016). For single-pulse labeling, a single dose of 10 mg/kg of animal weight of EdU (10 mg/mL in water) was administered intra-peritoneally.

#### Retrograde labeling

Anesthetized pups were placed in a stereotaxic apparatus at postnatal day (P) 9 and injected with red Retrobeads (Rbeads) IX from Lumafluor in the primary somatosensory cortex (S1) (200 nL; coordinates from the lambda: anteroposterior: 3 mm, mediolateral: 3 mm).

#### Tissue preparation

Embryos were collected 24, 48 and 72 hrs following *in utero* electroporation and post-fixed overnight in 4% paraformaldehyde (PFA, Sigma) at 4°C. Postnatal mice from P0 were perfused intracardially with 4% PFA and post-fixed overnight in 4% PFA at 4°C.

#### Immunohistochemistry and imaging

80 μm coronal sections were performed using a vibratome (Leica, #VT100S). Sections were permeabilized in PBST (0.3% Triton X-100, diluted in PBS 1X) and incubated for two hrs at room temperature in blocking solution (10% Horse Serum Albumine in PBST), then overnight at 4°C with primary antibodies. Treatment with HCl 2N at 37°C for 30′ was performed before incubation with standard blocking solution for KI67 immunohistochemistry. Treatment with Na citrate pH 6 at 80°C for 40′ was performed before incubation with standard blocking solution for RORB immunohistochemistry.

Sections were rinsed three times in PBST and incubated for 2 hrs at room temperature with corresponding secondary antibodies (1:500, Life Technologies). Three washes in PBST were performed, followed by 10 min incubation with Hoechst staining solution (1:5000 in PBS 1X, Life Technologies) to label nuclei, before dry mounting on slides with Fluoromount (Sigma). For imaging, the putative primary somatosensory cortex (S1) was used as region of study for all the experiments. Images were acquired on Eclipse 90i epifluorescence microscope (Nikon) or on LSM 700 confocal laser scanning microscope (Carl Zeiss).

#### Antibodies

Chicken anti-GFP (1:2000; Abcam, #AB13970); Goat anti-TOM (1:300; Sicgen, #AB8181-200); Rabbit anti-RFP (1:100; Abcam, #AB62341); Mouse anti-PAX6 (1:300; Thermoscientific, #MA1-109); Rat anti-EOMES (1:500; Invitrogen, #14-4875-82); Rat anti-EOMES (1:300; eBioscience, #144875-82); Rabbit anti-KI67 (1:250; Abcam, #AB15580); Rabbit anti-NEUROD2 (1:1000; Abcam, #AB104430); Mouse anti-RORB (1:200; Perseus Proteomics, #PP-N7927-00); Rabbit anti-CUX1 (1:250; Santa Cruz, #sc13024), Rabbit anti-CASPASE3 (1:3000; Cell signaling technology, #9662S), Mouse anti-BRN2 (1:200; Santa Cruz, #sc393324). All secondary antibodies were 488/555/647 conjugated (1:500, Invitrogen).

#### Time-lapse imaging

Acute slices were prepared at E17.5 from brains electroporated into the dorsal pallium at E14.5 with either *pSilencer scram*, *pSilencer miR-137* or *pSilencer miR-122* along with *pUb6-tdTOM* (reporter) plasmids. Briefly, brains were dissected out in ice-cold HBSS (Gibco), embedded in 3% LM-agarose (Roth) and cut 250 μm-thick with a vibratome (Leica VT1000S). Slices were then transferred on Millicell inserts (Merck Millipore) on NBM medium in incubator at 37°C for at least 2h recovery before being placed in a Fluorodish (WPI) for imaging. In all the experiments, scramble and miR-electroporated cell were imaged simultaneously. Statistical significance was set at α < 0.05. All calculations were done using Excel (version 16.31) and statistics were done with GraphPad Prism software (version 8.1.2). Normality of the samples was assessed with D'Agostino-Pearson test and non-parametric tests used when criteria were not fulfilled.

##### Multipolar-bipolar transition and radial migration imaging

Imaging was achieved with a live confocal microscope (Nikon A1r) equipped with long-working distance 20x or 40x objectives (0.45 and 0.6, respectively, CFI ELWD Plan Fluor, Nikon). The microscope chamber was kept at 37°C, with 25L/h continuous flux of 5% CO_2_ humidified at 95%. 50μm-thick z-stacks (3μm-stepped) were acquired with resonant laser scanning every 10min for 12h. In all the experiments, scramble and miR-electroporated cells were imaged simultaneously. At the end of the imaging session, stacks were piled into maximal intensity projections and time sequences corrected for drift with ImageJ (StackReg). To measure the multipolar to bipolar switch (Figure 4F), a box of 300 × 150μm was drawn at the transition zone with the upper border aligned on the IZ/deep layers boundary. All multipolar-shaped cells contained in this box were followed along 12 h and the percentage of cells transiting from multipolar morphology to bipolar shape was calculated. Cell movements were manually tracked and dynamic data extracted with ImageJ (MTrackJ). A cell was considered pausing when its movement along 10 min was under 12μm. Average speed is the distance achieved by a cell divided by the time taken to travel it, movement speed is the same calculation but not considering the events of pausing and directionality is the distance in straight line between the start and endpoint of a cell divided by the distance it traveled between these two points (Figures 4F and S4B). After manual tracking and data extraction, a random selection of an equal number of cells per slices was applied to avoid overrepresentation bias. All calculations were done using Excel (version 16.31) and statistics were done with GraphPad Prism software (version 8.1.2), with statistical significance set at α < 0.05. Normality of the samples was assessed with D'Agostino-Pearson test and non-parametric tests used when criteria were not fulfilled.

#### Tissue microdissection, cell sorting and RNA sequencing

##### Embryonic cortex

Tissue collection was performed as in Telley et al., 2019. Briefly, pregnant females were sacrificed, and embryos at E15.5 (*n* = 6 Scramble and *n* = 6 miR-137 embryos) and E17.5 (*n* = 6 Scramble and *n* = 6 miR-122 embryos) after E14.5 *in utero* electroporation extracted in ice-cold HBSS. 300μm-thick acute coronal brain sections were cut after embedding in 4% low melt-agar using a vibratome (Leica, #VT1000S) under RNase-free conditions. The putative S1 was microdissected using a Dissecting Scope (Leica, #M165FC) and incubated in 0.05% trypsin at 37°C for 5 minutes. Following tissue digestion, cells were incubated in fetal bovine serum and manually dissociated via gentle pipetting. Cells were then centrifuged for 5 minutes at 500 rpm, resuspended in 1 mL of HBSS, filtered using a 70 μm-pored cell strainer (ClearLine, #141379C) and incubated for 10 minutes at 37°C with Hoechst (0.1 mg/mL).

##### Postnatal cortex

Tissue collection was performed as in (Klingler et al., 2019). Briefly, 300 μm acute coronal brain sections were cut on a vibratome and S1 was microdissected as described above in ice-cold oxygenated artificial cerebrospinal fluid (ACSF) under RNase-free conditions.

P3: *n* = 4 E14 GFP electroporated pups; *n* = 4 E15.5 GFP electroporated pups. P7: *n* = 4 E14 GFP electroporated pups; *n* = 4 E15.5 GFP electroporated pups; *n* = 4 E14.5 Scramble + GFP electroporated pups; *n* = 4 E14.5 miR-122 + GFP electroporated pups; *n* = 4 E14.5 miR-137 + GFP electroporated pups.

For L4 *vs*. L2/3 comparison at P3 and P7 (i.e. E14 *vs*. E15.5 GFP electroporated pups), layers were microdissected following the same procedure as described above with further separation of superficial (SL) and deep (DL) layers. 5000 to 10′000 SL and DL cells were next dissociated by incubating microdissected samples in 0.5 mg/mL Pronase (Sigma, #P5147) at 37°C for 10 minutes, followed by a 3 minute inactivation in 5% bovine serum albumin, washes in ACSF and manual trituration using pulled glass pipettes of decreasing diameters. Cells were then centrifuged for 10 minutes at 500 rpm, resuspended and filtrated using a 70 μm- cell strainer (ClearLine, #141379C) and incubated for 10 minutes at 37°C with Hoechst (0.1 mg/mL).

Singlet GFP^+^/Hoechst^+^ embryonic and postnatal cells were sorted using a Beckman Coulter Moflo Astrios FAC-sorter according to their Forward and Slide scattering properties, and their negativity for Draq7™ (Viability dye, far red DNA intercalating agent, Beckman Coulter, #B25595). 5000 to 10′000 cells were FAC-sorted for each experiment. 3 μL of C1 Suspension Reagent (Fluidigm) was added to 10 μL of FACsorted cells, which were captured into 800 well- AutoPrep integrated fluidic circuit (IFC) designed for 10 to 17 μm diameter-cells (Fluidigm HT800, #101–4982) for embryonic cells, and for 10 to 17 μm diameter-cells (Fluidigm HT800, #100-57-80) for postnatal cells, and imaged using the ImageXpress Micro Widefield High Content Screening System (Molecular Devices®). cDNA synthesis and preamplification was processed following the manufacturer's instructions (C1 system, Fluidigm). cDNA libraries were prepared using Nextera XT DNA library prep kit (Illumina), quality control was done using 2001 Bioanalyzer from Agilent and sequenced using HiSeq 2500 sequencer for E14.5 electroporated pups, and HiSeq 5000 sequencer for E14 and E15.5 electroporated pups.

##### Bulk RNA sequencing

Brains were collected from E14.5 *in utero* electroporated miR-122 pups at P7 (*n* = 3 pups). S1 was microdissected following the same procedure as described above with further separation of superficial (SL) and deep (DL) layers. 5000 to 10’000 SL and DL cells were dissociated and FAC-sorted as for single-cell RNA sequencing. RNA from each sample was extracted using Total RNA Isolation System kit (Promega SV) and quality control was done using 2001 Bioanalyzer from Agilent. cDNA libraries were obtained using SMARTseq v4 kit (Clontech, # 634888) and sequenced using HiSeq 2500 sequencer. All single cell RNA capture, library preparations and sequencing experiments were performed at the Genomics Core Facility of the University of Geneva.

#### Electrophysiology

In *utero* electroplated (at E14.5) CD1 mice from P20-P26 were used for electrophysiological recordings. Mice were anesthetized with isoflurane and decapitated to dissect out the brains and were immediately transferred into ice-cold sucrose cutting solution equilibrated with 95% O_2_ and 5% CO_2_ containing (in mM) Sucrose (75), NaCl (85), CaCl_2_ (0.5), MgCl_2_ (4), NaHCO_3_ (24), KCl (2.5), NaH_2_PO_4_ (1.25) and glucose (25). Three hundred μm thick coronal slices were cut using a vibratome (Leica VT 1200S).

For another set of experiments, embryonic slices were obtained at E17.5 from electroporated embryos (E14.5). Briefly, embryos were surgically dissected and transferred in ice-cold HBSS (Gibco, 14175-053), embedded in 2% agarose solution, and four hundred μm thick coronal slices were cut in HBSS using a vibratome (Leica VT 1200S).

Slices were incubated at 35 °C for 20 min in a slice recovery chamber filled with artificial cerebrospinal fluid (ACSF) containing (in mM) NaCl (125), CaCl_2_ (2.5), MgCl_2_ (1), NaHCO_3_ (26), KCl (2.5), NaH_2_PO_4_ (1.25) and glucose (25). Slices were kept at room temperature in a recovery chamber until recording. For recording, slices were transferred to a recording chamber continuously perfused with oxygenated ACSF that was maintained at 30 ± 0.1°C (34 ± 0.1°C, for embryonic slices) using an in-line heating system (TC-01, Multichannel systems). All the recordings were carried out into the somatosensory cortex; the barrels in L4 were used as a visual landmark for identification. Embryonic recordings were carried out in intermediate zone. Cortical layers 2/3 and 4 neurons were visualized by using an upright microscope and camera system (BX51WIF, Olympus, and SciCam Pro CCD camera, Scientifica), equipped with a 40x water-immersion objective, infrared/differential interference contrast (DIC) optics, and epifluorescence (GFP filter set and a LED source: COO-LED2LLG-470-565, CoolLED). Electroporated neurons were identified using GFP expression and were used for further recordings. Whole-cell recordings were obtained with recording pipettes of resistance between 3 and 5 MΩ. The recording pipettes were pulled using borosilicate glass capillaries (1.5 mm OD, GC150TF-7.5, Harvard Instruments) on Zeitz DMZ puller (Zeitz instrument). Pipettes were filled with an internal solution containing (in mM) CH_3_KO_3_S (140), MgCl_2_ (2), NaCl (4), creatine phosphate (5), Na_2_ATP (3), GTP (0.33), EGTA (0.2) and HEPES (10) adjusted to 295 mOsm and pH 7.3 with KOH.

Whole-cell voltage-clamp recordings were obtained using a Multiclamp 700B amplifier (Axon Instruments) filtered at 3 kHz and digitized at 20 kHz (NI-6341, National Instruments Board and Igor, WaveMetrics). Neurons were held at −70 mV in voltage-clamp mode and a pulse of −4 mV was given at 0.1 Hz to monitor series resistance (R_s_). Neurons with a stable R_s_ and stable resting membrane potential (RMP) negative than −60 mV were subjected to a battery of current injection protocol to study electrophysiological properties. I_h_ currents were recorded in voltage-clamp using a - 40 mV step (500 ms) and were calculated by the difference of current between the beginning and the end of the voltage step. Spontaneous excitatory postsynaptic currents (sEPSCs) were recorded at V_h_ = −70 mV 1 μM of SR95531 hydrobromide (Tocris, Cat no. 1262) was bath applied to block inhibitory neurotransmission. Firing characteristics were studied in the current-clamp mode by injecting incremental steps of depolarizing currents from+50 pA to 500 pA for 500 ms.

#### Dual-Luciferase reporter assay

The cDNA from E14.5 mouse brains was used to amplify 3′UTR fragments of approximately 1000 bp containing one or multiple miRNA targeting sites. Amplified sequences were cloned by Gibson Assembly (NEB, E2611S) downstream of the luciferase gene in the pmirGLO Dual-Luciferase miRNA target expression vector (Promega, E1330).

HEK293T cells were seeded in 96-well plates (Corning, 3903) at a density of 35,000 cells in 50 uL culture medium (DMEM, 10% FBS, and 1% HEPES buffer) per well. Twenty-four hours later, cells were transiently transfected with pmiRGLO or pmiRGLO +3′UTR plasmids (40 ng/well) and miRNA delivering plasmids (60 ng/well) using Polyethylenimine (PEI) transfection (10 uL per well: 100 ng DNA, 0.7 uL of PEI 1g/mL, 2 uL of 1.5M NaCl, and ddH_2_O). Firefly Luciferase and Renilla luminescence were measured using Dual-Glo Luciferase Assay System (Promega, E2920) on an Infinite M1000Pro (Tecan) instrument.

### Quantification and statistical analysis

#### Histological analyses

Zen (Zeiss) and ImageJ softwares were used to analyze images. All results are shown as mean ± SEM, except when indicated otherwise. For statistical analyses, the following convention was used: ^∗^: p < 0.05, ^∗∗^: p < 0.01, ^∗∗∗^: p < 0.001. ‘‘Student's t-test’’ refers to the unpaired test. For embryonic analyses, differently electroporated embryos from the same mother were used to reduce plug timing variability. Experiments were cross-quantified blindly (i.e., the investigator was unaware to which of the experimental conditions the sections were belonging). Where indicated, scram, miR-137 and miR-122 were analyzed together in order to perform more robust statistical analysis (One-way Anova in place of Unpaired t Test).

Figures 1B–1D and 2D:Three sections for each brain electroporated with pUB6-TOM + pSilencer-U6-scram (number of brains, *n* = 3) or pUB6-TOM + pSilencer-U6-miR-137 (number of brains, *n* = 3) or pUB6-TOM + pSilencer-U6-miR-122 (number of brains, *n* = 3) at E12.5 or E14.5, were used to quantify the number of KI67^+^, NEUROD2^+^, PAX6^+^ and EOMES^+^, cells among the fraction of TOM^+^ cells 36, 48 or 72hr after IUE. Three sections for each brain electroporated with pSilencer-U6-scram + dUP-Cd63 (number of brains, *n* = 3) or pSilencer-U6-scram + dUP-Myt1l (number of brains, *n* = 3) at E14.5 were used to quantify the number of KI67^+^, NEUROD2^+^, PAX6^+^, EOMES^+^, cells among the fraction of GFP^+^ cells 48hr after IUE.

Figure 1B: percentage of VZ + SVZ KI67^+^ in E16.5 scram: 47.91 ± 1.6, E16.5 miR-137: 75.11 ± 3.12, E16.5 miR-122: 50.37 ± 2.31. Percentage of KI67^+^ in VZ E16.5 scram: 56.81 ± 1.67, VZ E16.5 miR-137: 34.69 ± 2.3, VZ E16.5 miR-122: 53.68 ± 1.17, SVZ E16.5 scram: 43.18 ± 1.67, SVZ E16.5 miR-137: 65.3 ± 2.3, SVZ E16.5 miR-122: 46.31 ± 1.17.

A one way-ANOVA with Dunnett's post hoc test was used when required.

Figure 1C: percentage of NEUROD2^+^ in E17.5 scram: 49.44 ± 1.24, E17.5 miR-137: 63.09 ± 1.4, E17.5 miR-122: 52.9 ± 0.67. A one way-ANOVA with Dunnett's post hoc test was used when required.

Figure 1D: ratio of PAX6^+^/EOMES^+^ in E16.5 scram: 1.03 ± 0.02, E16.5 miR-137: 3.76 ± 0.18, E16.5 miR-122 (not shown): 1.25 ± 0.07, E14 scram: 1.13 ± 0.18, E14 miR-137: 3.25 ± 0.33. A one way-ANOVA with Dunnett's post hoc test was used for E16.5. A student-T test was used for E14.

Figure 2D: percentage of KI67^+^ in E16.5 scram: 47.91 ± 1.6, E16.5 miR-137: 75.11 ± 3.12, scram + Myt1l-gfp: 34.85 ± 1.02, scram + Cd63-gfp: 73.31 ± 1.79. Percentage of NEUROD2^+^ in E16.5 scram: 49.46 ± 1.17, E16.5 miR-137: 35.14 ± 1.75, scram + Myt1l-gfp: 71.5 ± 2.26, scram + Cd63-gfp: 36.31 ± 1.52, E16.5 miR-137: 3.76 ± 0.18. Ratio of PAX6^+^/EOMES^+^ in E16.5 scram: 1.03 ± 0.02, E16.5 miR-137: 3.76 ± 0.18, scram + Myt1l-gfp: 1.1 ± 0.1, scram + Cd63-gfp: 2.05 ± 0.07, E16.5 miR-137: 3.76 ± 0.18. The same scram, miR-137 and miR-122 brains were used for the Figures 1B or 1C or 1D and 2D. Scram, miR-137, miR-122, scram + Myt1l and scram + Cd63 were analyzed together to minimize biological variability. A one way-ANOVA with Dunnett's post hoc test was used when required.

Figure S1E: three to four sections for each brain electroporated at E14.5 with DFRS or DFRS137 or DFRS122 and pSilencer-U6-miR-122 (number of brains, *n* = 3) or pSilencer-U6-miR-137 (number of brains, *n* = 3) were used to quantify the ratio of GFP^+^/RFP^+^ cells at E15.5. The analyses have been carried blindly. Ratio of GFP^+^/RFP^+^ in DFRS: 0.97 ± 0.08, DFRS137: 0.71 ± 0.03, DFRS137+pSil-miR-137: 0.25 ± 0.03, DFRS122: 0.42 ± 0.02, DFRS122+pSil-miR-122: 0.25 ± 0.002. A one way-ANOVA with Tukey's post hoc test was used when required.

Figures 3A and 5A: two to three sections for each electroporated brain were used to define the laminar position (Y coordinate) of electroporated cells at P7. Analyses were carried blindly. The Y value was normalized as percentage of distance from the WM. The cortex was divided in 10 bins and the mean value of frequency distribution was plotted in a bar graph. Dark-colored bins represent significant difference for the considered conditions. The Y values were plotted grouped by brain and the mean value and standard deviation were represented. Density plot and cumulative distribution were used to additionally display the Y values.

Figure 3A: IUE at E14.5 with pUB6-TOM and pSilencer-U6-scram (number of brains, *n* = 4) or pSilencer-U6-miR-137 (number of brains, *n* = 4). Mean value for Y position in E14.5 control: 36.13 ± 0.29, miR-137: 34.36 ± 0.27.

Figure 5A: IUE at E14.5 with pUB6-TOM and pSilencer-U6-scram (number of brains, *n* = 4) or pSilencer-U6-miR-122 (number of brains, *n* = 4). Mean value for Y position in E14.5 control: 36.13 ± 0.29, miR-122: 66.67 ± 0.79. The same scram brains were used for the Figures 3A and 5A. Scram, miR-137 and miR-122 were analyzed together to minimize biological variability. A two-way ANOVA with Bonferroni's post-hoc test was used for bin analysis; a one-way ANOVA was used for mean Y position.

Figure 3B: three sections for each brain electroporated at E14.5 with pUB6-GFP and pSilencer-U6-scram (number of brains, n = 4) or pSilencer-U6-miR-137 (number of brains, n = 4) and chronically-delivered EdU at E16.5, were used to quantify the number of EdU cells among the total amount of GFP^+^ cells in L2/3 at P7. Percentage of EdU/GFP^+^ scram: 10.74 ± 0.74, miR-137: 27.55 ± 2.09. A student-T test was used.

Figures 3D, 5C, and S3C: three sections for each brain electroporated at E14.5 with pUB6-GFP and pSilencer-U6-scram (number of brains, *n* = 4) or pSilencer-U6-miR-137 (number of brains, *n* = 4) or pSilencer-U6-miR-122 (number of brains, n = 4) were used to quantify the number of RORB^+^ cells among the fraction of electroporated cells in L2/3 and L4 at P7. Ratio of RORB^+^ in E14.5 L2/3 control: 0.06 ± 0.01, L2/3 miR-137: 0.07 ± 0.008, L2/3 miR-122: 0.03 ± 0.004, L4 control: 7.29 ± 0.46, L4 miR-137: 2.53 ± 0.23, L4 miR-122: 0.62 ± 0.11. A two-way ANOVA with Bonferroni's post-hoc test was used.

Figure S3D: L4 POU3F2 intensity of scram cells: (0.29 ± 0.01), miR-137 (0.47 ± 0.01). A one-way ANOVA with Tukey's post hoc test was used for the analysis. The fluorescence intensity (0–255 scale of 8-bit images) for POU3F2^+^ of the counted cells has been plotted to show the expression of the marker.

Figure 3E: coronal sections from at least 3 different brains electroporated at E14.5 with pUB6-GFP and pSilencer-U6-scram (total of 676 cells) or pSilencer-U6-miR-137 (total of 969 cells) were used to quantify the percentage of electroporated cells displaying an apical dendrite at P7 in L4. Percentage of cells with apical dendrite in E14.5 scram: 9.99 ± 1.33, E14.5 miR-137: 16.69 ± 1.58. A student-T test was used.

Figure 3G: three sections for each brain electroporated at E14.5 with pUB6-TOM and pSilencer-U6-scram (number of brains, *n* = 4) or pSilencer-U6-miR-137 (number of brains, *n* = 4) and injected with retrobeads (Lumafluor) at P9, were used to quantify the number of retrobeads^+^ cells among the fraction of TOM^+^ cells at P11. Ratio of retrobeads^+^ in L2/3 scram: 1 ± 0.14, L4 scram: 0.03 ± 0.01, L4 miR-137: 0.23 ± 0.09. A two-way ANOVA with Tukey's post-hoc test was used when required.

Figures 4E and S4C: three sections for each brain electroporated at E14.5 with pUB6-TOM or pUB6-GFP and pSilencer-U6-scram (number of brains, *n* = 3) or pSilencer-U6-miR-122 (number of brains, *n* = 3) or pSilencer-U6-miR-137 (number of brains, *n* = 3, not shown) were used to quantify the number of TOM^+^ or GFP^+^/SATB2^+^ cells in each cortical layer at E17.5. The analyses have been carried blindly.

Figure 4E: Distribution of scram cells: (VZ/SVZ: 15.05 ± 0.47, IZ: 50.92 ± 0.7, L5/6: 21.5 ± 0.25, SL: 12.51 ± 1.03), miR-122 (VZ/SVZ: 35.91 ± 1.71, IZ: 61.07 ± 1.48, L5/6: 1.86 ± 0.04, SL: 1.14 ± 0.25), miR-137 (VZ/SVZ: 8.67 ± 1.06, IZ: 44.21 ± 1.23, L5/6: 17.66 ± 0.84, SL: 29.45 ± 1.9). A two-way ANOVA with Sidak's post hoc test was used.

Figure S4C: SATB2 intensity of scram cells: (Global: 0.48 ± 0.04, SVZ: 0.11 ± 0.02, IZ: 0.47 ± 0.04, L5/6: 0.59 ± 0.05, SL: 0.61 ± 0.06), miR-122 (Global: 0.28 ± 0.02, SVZ: 0.09 ± 0.02, IZ: 0.29 ± 0.02, L5/6: 0.33 ± 0.02, SL: 0.45 ± 0.02). A student-T test was used for the global analysis. A two-way ANOVA with Sidak's post hoc test was used for the layer-wise analysis. The fluorescence intensity (0–255 scale of 8-bit images) for SATB2^+^ of the counted cells has been plotted to show the expression of the marker.

Figure 5E: three sections for each brain electroporated at E14.5 with pUB6-GFP and pSilencer-U6-scram (number of brains, *n* = 3) or pSilencer-U6-miR-122 (number of brains, *n* = 3) were used to quantify the number of GFP^+^ cells in each cortical layer at P21. Analyses were carried blindly. Distribution of scram cells: (L2/3: 40.63 ± 2.85, L4: 57.95 ± 2.34, L5/6: 1.42 ± 0.52, WM: 0), miR-122 (L2/3: 55.81 ± 0.25, L4: 37.74 ± 0.74, L5/6: 6.14 ± 0.69, WM: 0.29 ± 0.29). A two-way ANOVA with Sidak's post hoc test was used.

Figure S5A: three sections for each brain electroporated at E14.5 with pUB6-TOM with pSilencer-U6-scram (number of brains, *n* = 3) or pUB6-GFP with pSilencer-U6-miR-122 (number of brains, *n* = 3) were used to quantify the number of TOM^+^/EdU^+^ or GFP^+^/EdU^+^ cells in each SL or DL + WM at P7. Analyses were carried blindly. Distribution of scram cells: (SL: 99.94 ± 0.05, DL + WM: 0.05 ± 0.05), miR-122 (SL: 86.62 ± 0.63, DL + WM: 13.37 ± 0.63). A two-way ANOVA with Sidak's post hoc test was used.

Figure S5E: three sections for each brain electroporated at E14.5 with pUB6-TOM and pSilencer-U6-scram (number of brains, *n* = 3) or pUB6-TOM/pUB6-GFP and pSilencer-U6-miR-122 (number of brains, *n* = 3) were used to quantify the number of CASP3^+^ cells at P7. Analyses were carried blindly. Percentage of scram cells: (1.67 ± 0.98), miR-122 (0.51 ± 0.51). An Unpaired t Test was used.

Figure S5F: L4 POU3F2 intensity of scram cells: (0.29 ± 0.01), miR-122 (0.26 ± 0.01). A one-way ANOVA with Tukey's post hoc test was used for the analysis. The fluorescence intensity (0–255 scale of 8-bit images) for POU3F2^+^ of the counted cells has been plotted to show the expression of the marker.

Figure S5G: CUX1 intensity of scram cells: (5.48 ± 0.14), miR-122 (3.32 ± 0.14), miR-137 (9.15 ± 0.14, not shown). A two-way ANOVA with Sidak's post hoc test was used for global analysis. The fluorescence intensity (0–255 scale of 8-bit images) for CUX1^+^ of the counted cells has been plotted to show the expression of the marker.

#### Single-cell transcriptomic analyses

Reads were mapped on mouse genome GRCm38 following the same pipeline described in (Telley et al., 2019). Read 1, which contains the UMI sequence, was appended at the end of read 2 header; reads 2 were further mapped to the mouse genome with Tophat v2.0.13. Resulting alignment files in BAM format were processed with umi_tools (Smith et al., 2017) to deduplicate reads with identical UMI. Gene expression quantification was performed with R using summarizeOverlAP method of package GenomicAlignments. Only reads falling into exonic part of a gene are quantified (including 5′ AND-3′ UTRs).

For single-cell RNA sequencing, each transcriptome was further associated to a manual brightfield picture annotation, where the presence of a single cell in the wells of the Fluidigm HT800 chips was checked. Only wells where a single GFP^+^ cell was observed were kept for further analyses (wells with no cell, cell(s) with convoluted shapes, multiple cells, or cell(s) with debris were excluded).

All bioinformatics transcriptomics analyses were performed using R programming language and Bioconductor packages as described below on reads per million (RPM) normalized (log_10_) gene expressions.

#### Single cell filtering

*Embryonic single cells*: Cells expressing less than 2000 genes, or more than 15% mitochondrial genes were excluded from the analysis (resulting in E15.5: *n* = 192 scramble cells, *n* = 222 miR-137 cells; E17.5: *n* = 131 scramble cells, *n* = 204 miR-122 cells).

*Postnatal single cells*: Cells expressing less than 1000 genes, or more than 15% mitochondrial genes were excluded from the analysis (resulting in E14.5 *in utero* electroporation, P7: *n* = 341 P7 scramble cells, *n* = 352 miR-137 cells, *n* = 269 miR-122 cells; E14 *in utero* electroporation: P3: *n* = 207 scramble cells, P7: *n* = 162 scramble cells; E15.5 *in utero* electroporation, P3: *n* = 211 scramble cells, P7: *n* = 229 scramble cells).

*Clustering analysis of embryonic single cells*: Seurat bioinformatics pipeline (https://cran.r-project.org/web/packages/Seurat/citation.html) was used to determine the most 2000 variable genes using the FindVariableFeatures function (selection method = vst) and to perform t-Distributed Stochastic Neighbor Embedding dimensional reduction (tSNE) from the top 10 principal components. The FindClusters function (resolution = 0.5) next revealed 4 clusters, which were assigned to apical progenitor (AP), basal progenitor (BP), immature neuron (N_0_), and mature neuron (N_1_) identities based on their gene expression analyzed using the FindAllMarkers function. Expression of cell type-specific transcripts described in Telley et al., Science 2019 was used to further validate the identified clusters (Figures 2A and 4A).

*Pseudotime analysis of embryonic single cells* was performed for E17.5 scramble *vs*. miR-122 (Figure 4D) experiments using a regularized ordinal regression method (*bmrm* R package) to predict differentiation status of each cell. The linear models were built using the scramble cells, ranking the genes according to their ability to predict each cell category (AP, BP, N_0_ and N_1_; for E17.5 experiment, AP and BP were pooled together as they represent a very small cluster), and re-optimized on the best 100 genes (top 50 and bottom 50), before cross-validations using leave-one-out method. Pseudo-differentiation prediction scores were then calculated for miR or human embryonic neurons from Nowakowski et al., 2017 (Figures 4H and S4D; annotations from the original publication were used to define immature and mature neurons) cells by the linear combination of the top gene expression and compared to the cross-validation values of scramble cells.

*Predictions of SL vs. DL miR-122 identity* (Figure S5B)*, L2/3 vs. L4 identity* (Figures 3C, 5B, and S3A) *or P3 vs. P7 L2/3 (or L4) identity* (Figure S5C): A logistic regression model with regularization was used to build binary prediction models of: (1) microdissected SL *vs*. DL miR-122 cells (bulk RNA sequencing), (2) L2/3 (E15.5 *in utero* electroporated) *vs.* L4 (E14 *in utero* electroporated) P7 scram cells (single cell RNA sequencing), or (3) P3 *vs.* P7 L2/3 or L4 scram cells (single cell RNA sequencing). This implementation was provided by the hingeLoss function of the *bmrm* R package. This allowed to rank the genes based on their ability to predict the identity, and to re-train a new model on the best 100 genes (top- and bottom- 50 genes). All model performances were addressed by leave-one-out (1) or 20-fold (2, 3) cross-validations on the subset of genes, which gave a prediction value to build receiver operating characteristic (ROC) curves. The layer/age prediction scores were then calculated for E14.5 *in utero* electroporated P7 miR *vs*. scram cells by the linear combination of the top gene expression. Figures 3C and 5B: percentage of scram cells: (L2/3: 53.07, L4: 46.92), miR-122SL (L2/3: 80.37, L4: 19.62), miR-122DL (L2/3: 66.66, L4: 33.33), miR-137 (L2/3: 66.19, L4: 33.8). A Fisher's chi-square test was used.

*Differential gene expression analyses* between scram and miR-137 (E15.5: Figure 2, P7: Figure 3) or scram and miR-122 (E17.5: Figure 4, P7: Figure 5) cells were performed using logistic regression models for each cell type (hingeLoss function; *bmrm* R package). This allowed to rank the genes based on their ability to predict scram *vs*. miR identities, and to calculate an identity score based on the best 100 genes by 20-fold cross-validations. The areas under the ROC curve (AUC) were calculated based on the sensitivity/specificity of each model using the cross-validation values (roc.stat function).

All gene ontologies were performed using GSEA (Subramanian et al., 2005).

#### Electrophysiology data analysis

Neurons with R_s_ fluctuation of >20% during recording were excluded from the analysis. Offline analysis of electrophysiological data was carried out using Igor pro (Wave metric), several electrophysiological parameters were manually computed as illustrated in Figure S5D. The first AP elicited in response to threshold depolarizing current injection was used to calculate the single AP parameters. AP train elicited in response to the current injection of +500 pA for 1000 ms was used for calculation of spike frequency and other AP train parameters. For calculation of membrane properties, at least 12 consecutive sweeps were digitally averaged. The membrane time constant (tau) was computed by monoexponential fit to the first 100 ms after the current injection of −40 pA. sEPSCs were detected and analyzed using Mini Analysis Program (Synaptosoft) script integrated into Igor pro. Prism 8 (GraphPad) was used for statistical analysis and preparation of the graphs. All the data are presented as mean ± SEM.

Figure 3F: Coronal sections from at least 3 different brains electroporated at E14.5 with pUB6-GFP and pSilencer-U6-scram L2/3 (total of 13 cells) or L4 (total of 19 cells) or pSilencer-U6-miR-137 (total of 13 cells) were used to quantify the I_h_ current. Mean of cells with I_h_ in L2/3 scram: 59.85 ± 11.2, L4 scram: 27.32 ± 4.69, L4 miR-137: 43.53 ± 6.25. A student-t test was used.

## Data Availability

• All scRNA sequencing data are available in GEO: GSE159596 (accession number). All the other data generated in this study will be available upon request. For further inquiries, please address to the corresponding author or to the lead contact. • This paper does not report original code. • Any additional information required to reanalyze the data reported in this work paper is available from the Lead Contact upon request.

## Supplemental information

Document S1. Figures S1–S5

Document S2. Article plus supplemental information
