# Supplementary material for: miR-137 and miR-122, two outer subventricular zone non-coding RNAs, regulate basal progenitor expansion and neuronal differentiation
Source: Cell Rep. 2022 Feb 15;38(7):110381. doi: 10.1016/j.celrep.2022.110381 (PMC8864305; doi:10.1016/j.celrep.2022.110381)
Supplement: Document S1. Figures S1–S5 [file mmc1.pdf]

Cell Reports, Volume *38*

Supplemental information

miR-137 and miR-122, two outer subventricular zone non-coding RNAs, regulate basal progenitor expansion and neuronal differentiation

Ugo Tomasello, Esther Klingler, Mathieu Niquille, Nandkishor Mule, Antonio J. Santinha, Laura de Vevey, Julien Prados, Randall J. Platt, Victor Borrell, Denis Jabaudon, and Alexandre Dayer

Figure S1: microRNAs 137 and 122 expression level across species (related to Figure 1).

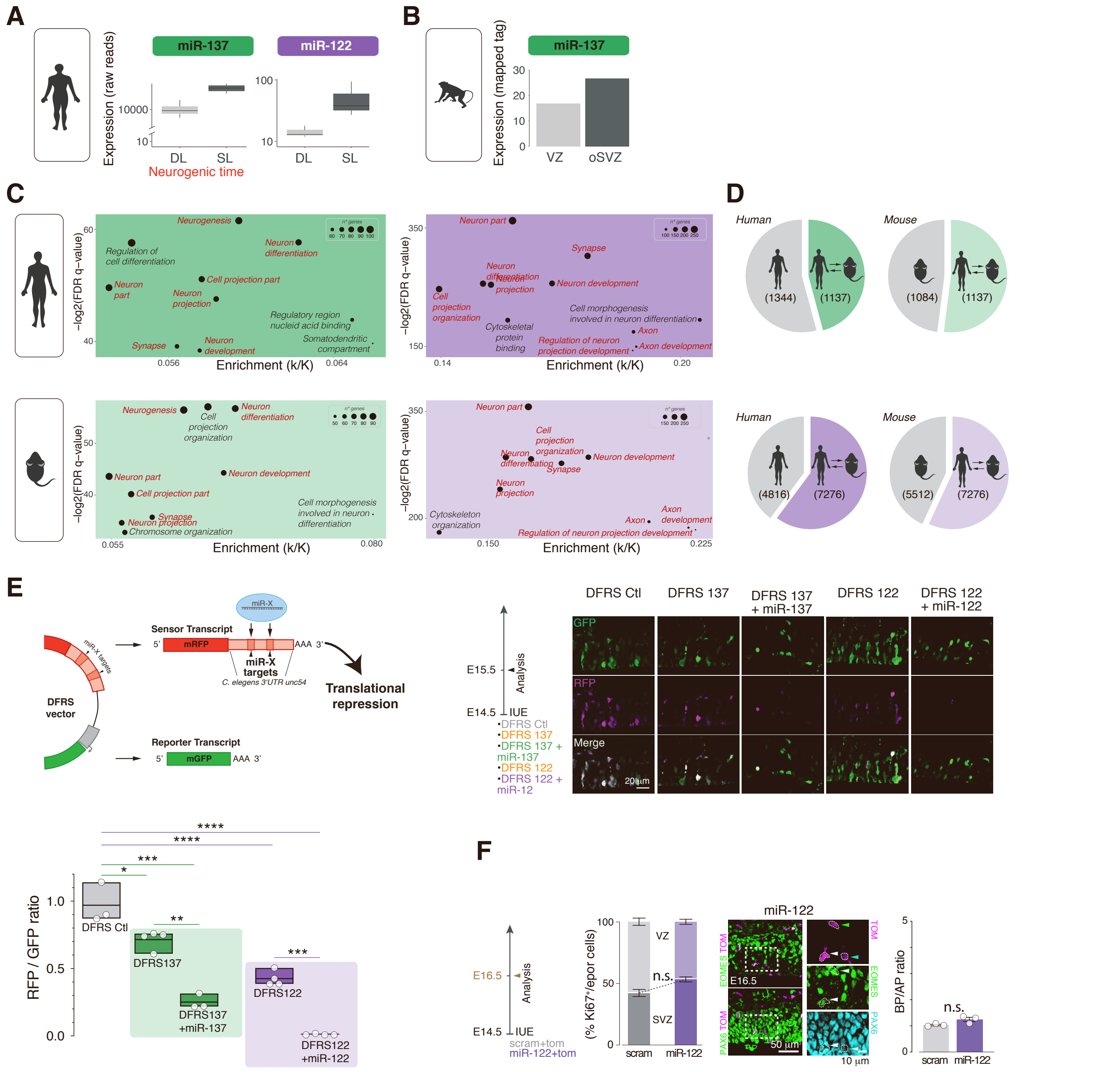

Figure S1: MicroRNAs 137 and 122 expression level across species.

(**A**) MiR-137 and miR-122 expression during superficial (SL, GSW 19-20) and deep (DL, GSW 15-16) layer neurogenesis in human. Data are from Nowakowski et al., 2018. (**B**) MiR-137 expression in VZ and oSVZ of macaque cortex during superficial layer neurogenesis. Data are from Arcila et al., 2014. (**C**) Gene ontologies of miR-137 (left) and miR-122 (right) predicted targets in human and mouse. Shared ontologies across species are in red. (**D**) Shared genes of miR-137 (left) and miR-122 (right) predicted targets in human and mouse. Values are indicated in brackets. (**E**) Endogenous expression of miR-137 and miR-122 in E15.5 mouse cortex assessed with DFRS 137 and DFRS 122, respectively (see Methods). Validation of miR-137 (DFRS 137 + miR137) and miR-122 (DFRS 122 + miR122) overexpression tools (DFRSCtl, n=3; DFRS137, n=4; DFRS137+miR-137, n=3; DFRS122, n=4; DFRS122+miR-122, n=4). (**F**) Progenitors in the VZ and SVZ at E16.5 upon miR-122 overexpression in E14.5 mouse cortex. Left, quantifications of KI67$^+$ electroporated cells in VZ and SVZ (scram and miR-122, n=3 each). Middle, representative micrographs of EOMES / PAX6 labelings. Green arrowheads, EOMES$^+$ electroporated cells; cyan arrowheads, PAX6$^+$ electroporated cells, white arrowheads, EOMES$^+$/PAX-6$^+$.Right,Basal progenitor (BP) versus apical progenitor (AP) ratio upon miR-137 overexpression at E14.5 addressed using immunohistochemistry against EOMES to identify BP and PAX6 to identify AP (scram and miR-122, n=3 each). Data are represented as mean ± SEM. (D, E left) One-way ANOVA. (E right) Unpaired t-test. Biological replicates are distinguished by circles in the bar plots. *$p < 0.05$, **$p < 10^{-2}$, ***$p < 10^{-3}$, ****$p < 10^{-4}$. Data are represented as mean ± SEM. (D, E left) One-way ANOVA. (E right) Unpaired t-test. Biological replicates are distinguished by circles in the bar plots. *$p < 0.05$, **$p < 10^{-2}$, ***$p < 10^{-3}$, ****$p < 10^{-4}$.

Figure S2: Transcriptional regulations upon miR-137 overexpression in basal progenitors. (related to Figure 2).

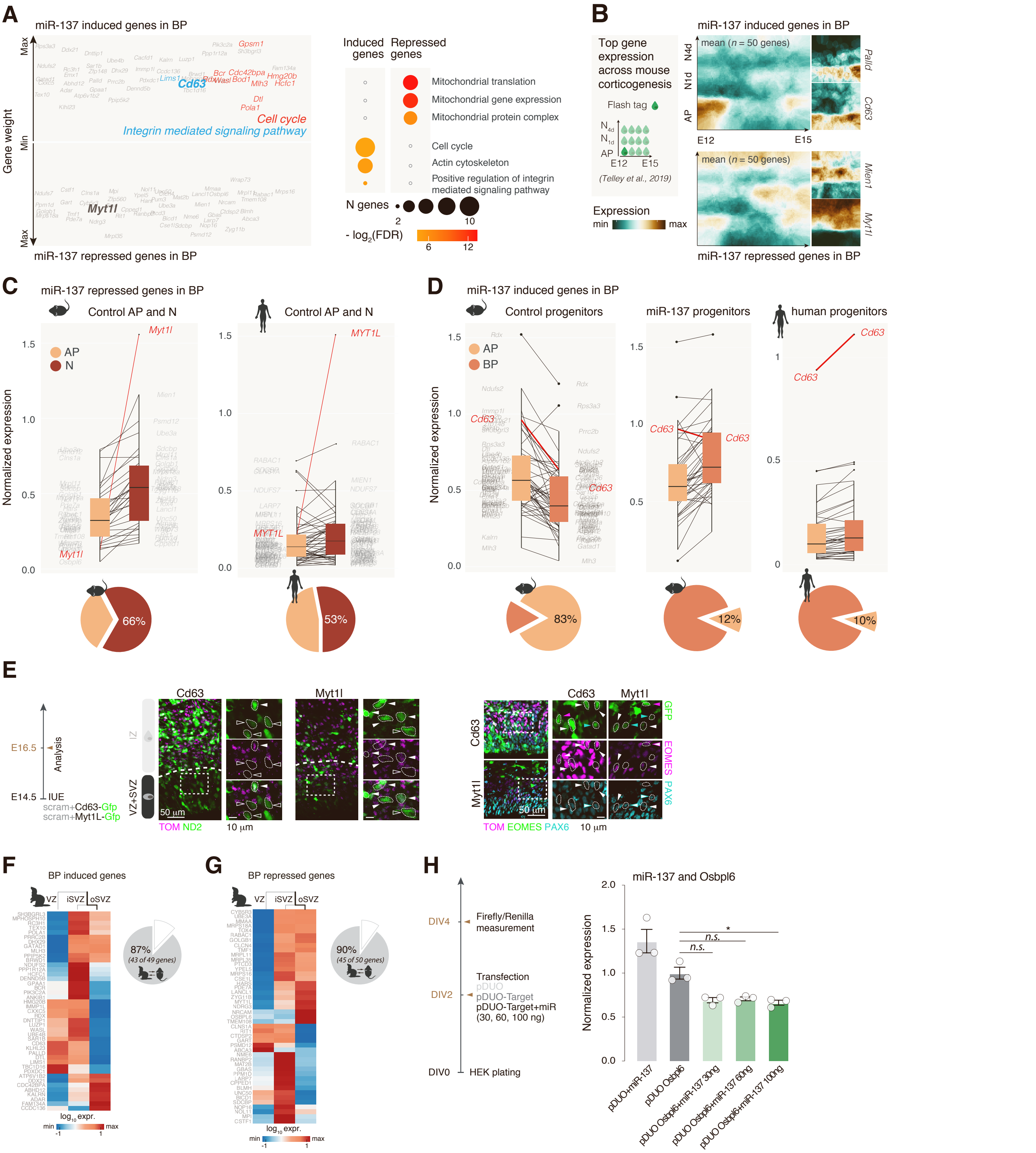

Figure S2: Transcriptional regulations upon miR-137 overexpression in basal progenitors. (**A**) Top 50 induced and repressed genes upon miR-137 overexpression in basal progenitors (BPs) at E15.5. Left, gene weights based on the support vector machine learning. Right, gene ontologies. (**B**) Expression of miR-137 induced and repressed genes in BPs in FlashTag labeled cells. Data are from Telley et al., 2019. (**C-D**) Expression of miR-137 repressed and induced genes in BPs in mouse and human control apical progenitors (AP) and neurons (N) (**C**), and in APs and BPs from control, miR-137 and human conditions (**D**), respectively. Pie charts represent the proportion of genes expressed in AP and N (C), and in AP and BP (**D**). Note that miR-137 induced genes in BPs are more expressed in control APs than BPs in mouse, while in both miR-137 and human conditions they are enriched in BPs. (**E**) Representative micrographs of NeuroD2 (left) and PAX6 / EOMES (right) immunohistochemistry performed after *Cd63* and *Myt1l* overexpression. In micrographs of EOMES / PAX6 labelings: magenta arrowheads, EOMES$^+$ electroporated cells; cyan arrowheads, PAX6$^+$ electroporated cells, white arrowheads, EOMES$^+$/-PAX6$^+$. (**F-G**) Expression of the top BP induced and repressed genes upon miR-137 overexpression in P2 microarray of ferret (data are from de Juan Romero et al., 2015). Left: heatmap of the BP induced or repressed genes expressed in ferret database. Right: piechart of BP induced or repressed genes shared between ferret and mouse. (**H**) Target validation of miR-137 by Luciferase assay. Left, experimental design. Right, quantification of luciferase activity (Firefly:Renilla ratio) normalized on control condition (reporter plasmid with recognition sequence for miR-137), (pDUO+miR-137, pDUO Osbpl6, pDUO Osbpl6+miR-137 30 ng, pDUO Osbpl6+miR-137 60 ng, pDUO Osbpl6+miR-137 100 ng, n=3 each). Data are represented as median ± SD or mean ± SEM (H). Biological replicates are distinguished by circles in the bar plots. One-way ANOVA (H). *p < 0.05, **p < 10^{-2}, ***p < 10^{-3}, ****p < 10^{-4}.

Figure S3: Layer 2/3 versus layer 4 molecular identity in miR-137 overexpressing neurons. (related to Figure 3).

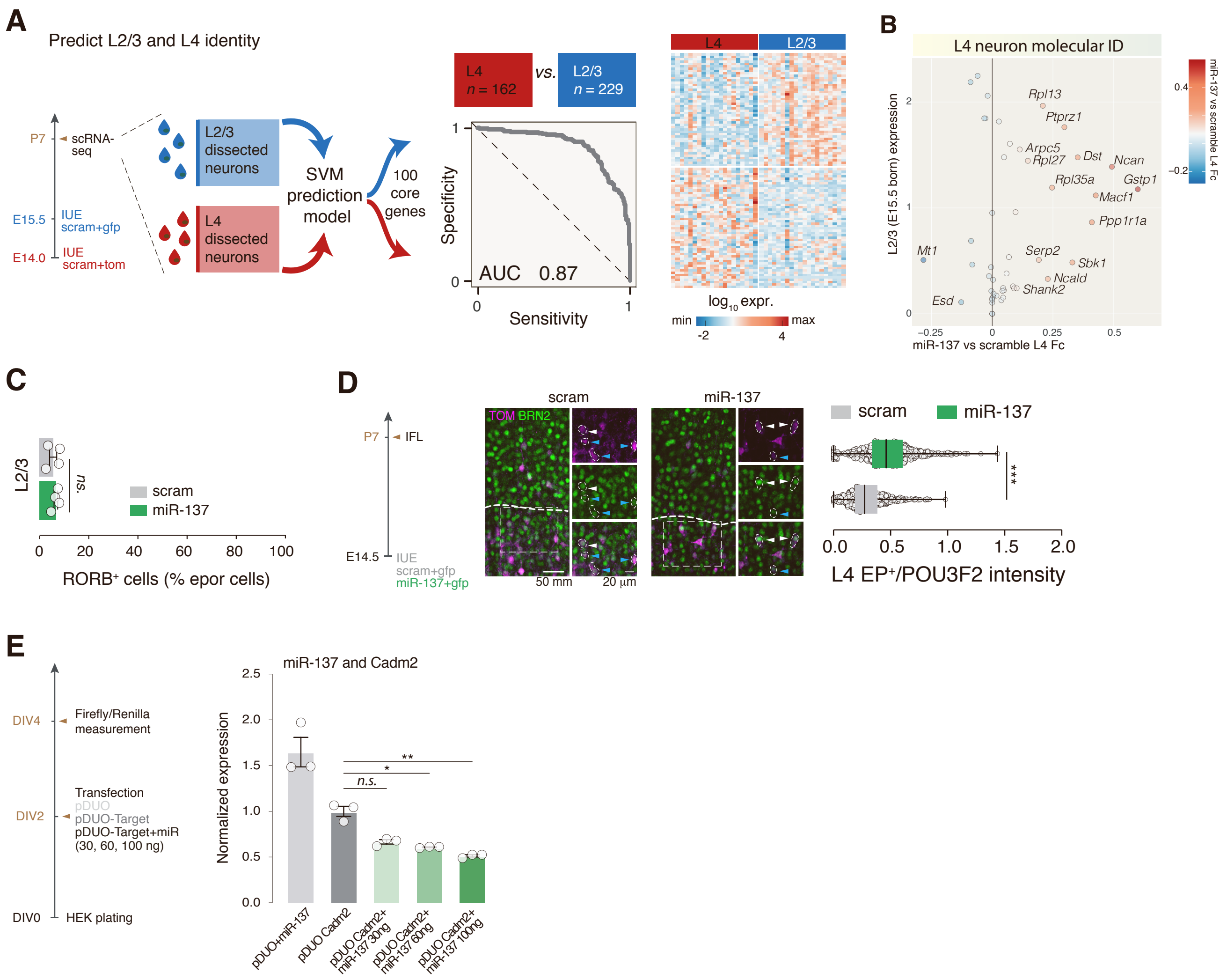

Figure S3. Layer 2/3 versus layer 4 molecular identity in miR-137 overexpressing neurons. (**A**) Left, schematic diagram of L2/3 and L4 P7 neuron isolation through birthdate-locked *in utero* electroporation (IUE). Middle, model performance using support vector machine learning approach to predict L2/3 *vs.* L4. Right, heatmap of top 100 genes which better defines L2/3 and L4 at P7. (**B**) Foldoing change expression of top 50 genes which defines L2/3 prediction, in L4 scram and miR-137 neurons. (**C**) RORB expression in L2/3 neurons in scram and miR-137 conditions (scram and miR-137, n=4 each). (**D**) BRN2 expression in L4 neurons in scram and miR-137 conditions (scram and miR-137, n=3 each). (**E**) Target validation of miR-137 by Luciferase assay. Left, experimental design. Right, quantification of luciferase activity (Firefly:Renilla ratio) normalized on control condition (reporter plasmid with recognition sequence for miR-137), (pDUO+miR-137, pDUO Cadm2, pDUO Cadm2+miR-137 30 ng, pDUO Cadm2+miR-137 60 ng, pDUO Cadm2+miR-137 100 ng, n=3 each). Data are represented as mean ± SEM. Biological replicates are distinguished by circles in the bar plots. Two-way ANOVA (C), One-way ANOVA (D, E). Biological replicates and recorded cells are distinguished by circles in the bar plots (C and D, respectively). $^{*}p < 0.05$, $^{**}p < 10^{-2}$, $^{***}p < 10^{-3}$, $^{****}p < 10^{-4}$.

Figure S4: MiR-122 controls the migration and maturation of differentiating neurons. (related to Figure 4).

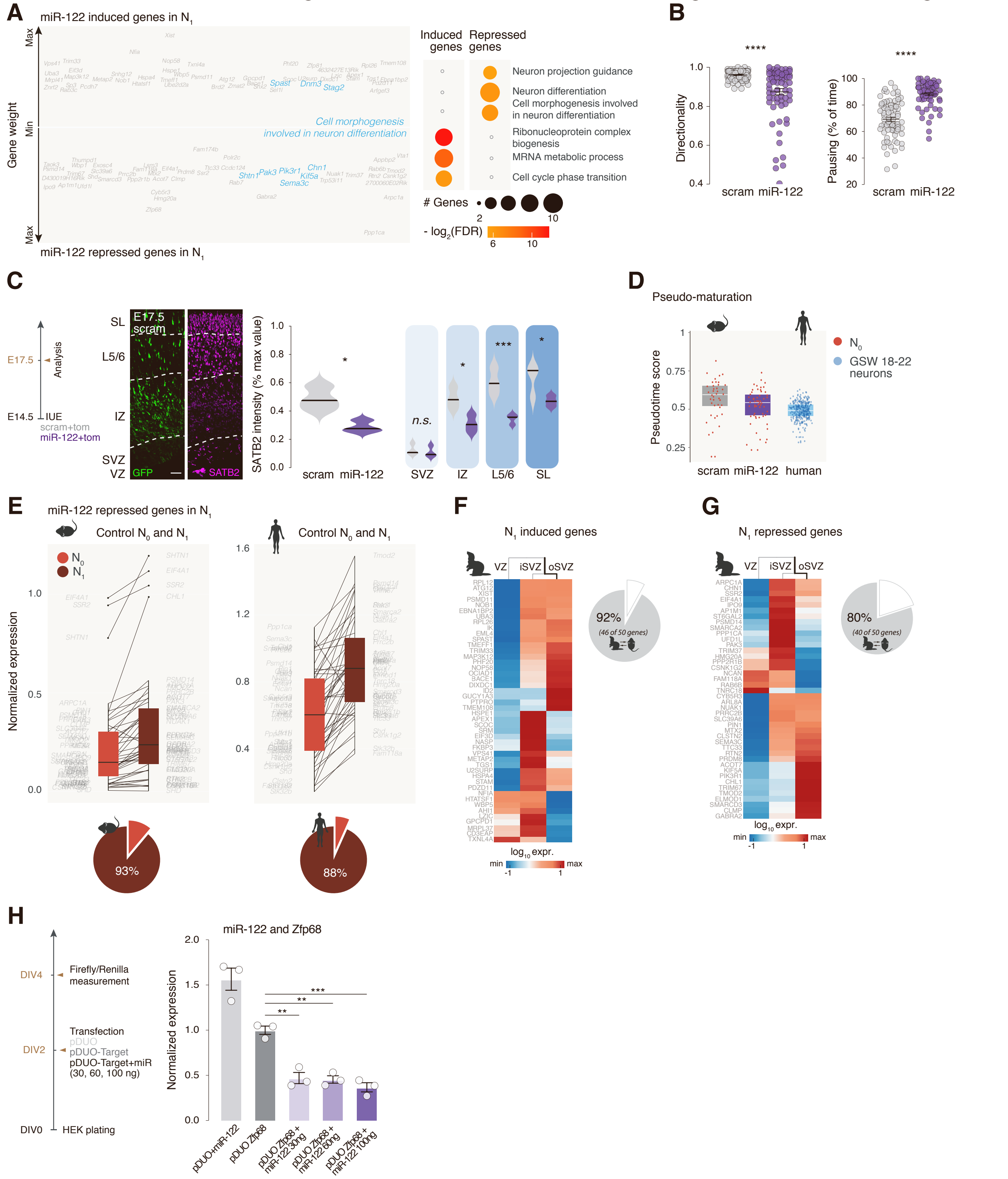

Figure S4: MiR-122 controls the migration and maturation of differentiating neurons.
(**A**) Top 50 induced and repressed genes upon miR-122 overexpression in differentiating neurons (N_1) at E15.5. Left, gene weights based on the support vector machine learning. Right, gene ontologies. (**B**) Directionality and pausing time of scrambled (scram) and miR-122 migrating neurons at E17.5 (scram, n=4, miR-122, n=3). (**C**) Expression of SATB2, a marker for SL mature neurons, in E17.5 scram and miR-122 neurons (scram and miR-122, n=3 each). (**D**) Pseudotime value predictions of human superficial layer immature neurons from gestational weeks (GSW) 18-22 foetuses using the mouse model. (**E**) Expression of miR-122 repressed genes in N_1s in mouse and human control newborn neurons (N_0) and immature neurons (N_1). Pie charts represent the proportion of genes expressed in N_0 and N_1. Note that miR-122 repressed genes in N_1s are more expressed in control N_1s than N_0s in mouse and in human conditions. (**F**) Expression of the top N_1 induced genes upon miR-122 overexpression in P2 microarray of ferret (data are from de Juan Romero et al., 2015). Left: heatmap of the N_1 induced genes expressed in ferret database. Right: piechart of N_1 induced genes shared between ferret and mouse. (**G**) Expression of the top N_1 repressed genes upon miR-122 overexpression in P2 microarray of ferret (data are from de Juan Romero et al., 2015). Left: heatmap of N_1 repressed genes expressed in ferret dataset. Right: piechart of N_1 repressed genes shared between ferret and mouse. (**H**) Target validation of miR-122 by Luciferase assay. Left, experimental design. Right, quantification of luciferase activity (Firefly:Renilla ratio) normalized on control condition (reporter plasmid with recognition sequence for miR-122), (pDUO+miR-122, pDUO Zfp68, pDUO Zfp68+miR-122 30 ng, pDUO Zfp68+miR-122 60 ng, pDUO Zfp68+miR-122 100 ng, n=3 each). Data are represented as mean ± SEM. Kruskal-Wallis (B); Unpaired t-Test (C, all layers); Two-way ANOVA (C, by layer); One-way ANOVA (H). *$p < 0.05$, **$p < 10^{-2}$, ***$p < 10^{-3}$, ****$p < 10^{-4}$. Human scRNA-seq data are from Nowakowski et al., 2017.

Figure S5: MiR-122 affects the laminar allocation and postnatal functional maturation of superficial layer cortical neurons. (related to Figure 5).

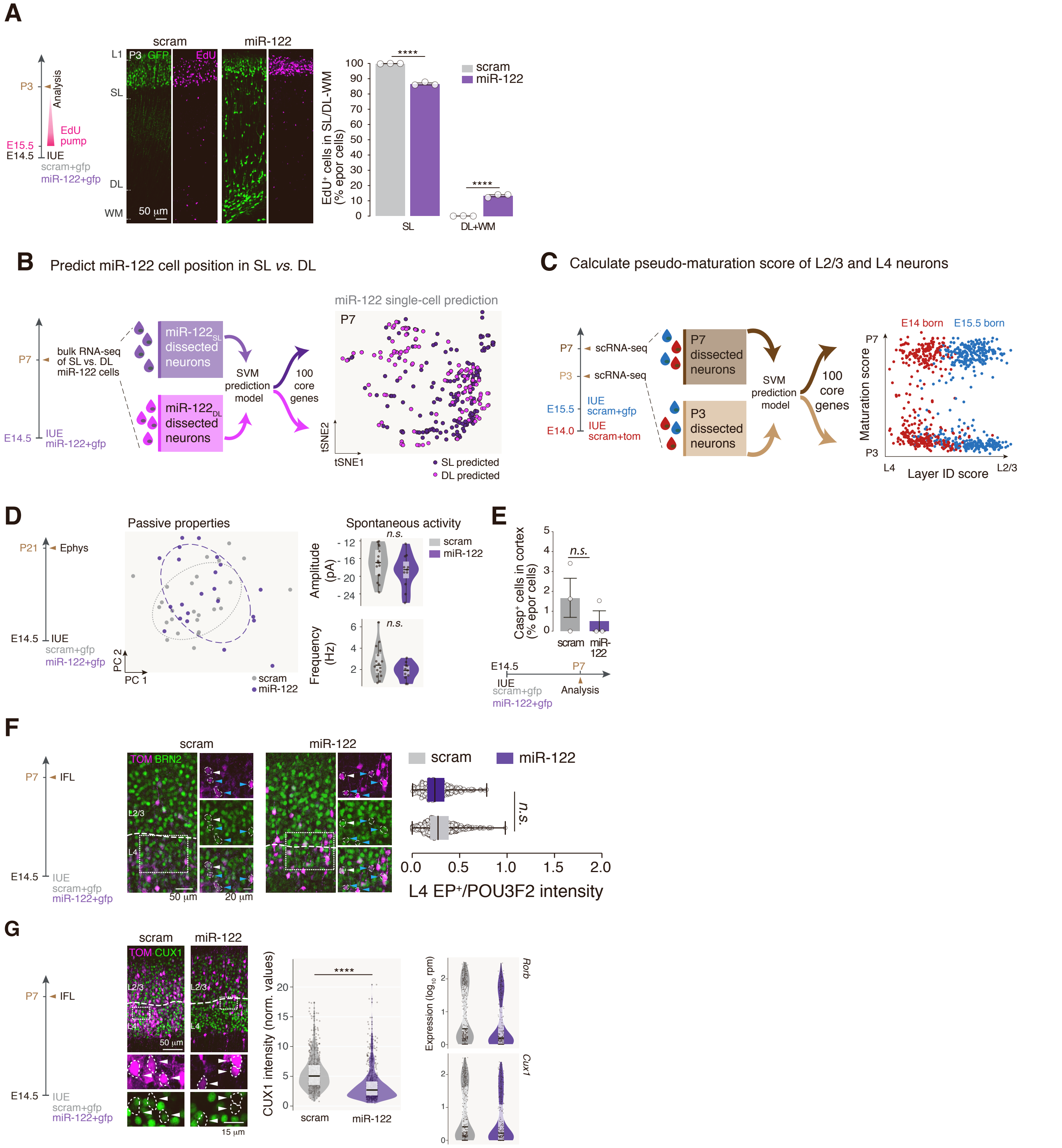

Figure S5: MiR-122 affects the laminar allocation and postnatal functional maturation of superficial layer cortical neurons. (**A**) Left, schematic diagram of the experiment to distinguish L2/3 neurons (born from E15.5 on) from L4 (born at E14.5) neurons after E14.5 *in utero* electroporations. Middle, EdU staining at P3. Right, Quantifications of EdU+ cells in superficial (SL) and deep (DL) layers/ white matter (WM) upon scram or miR-122 overexpression (scram and miR-122, n=3). (**B**) Left, schematic diagram of miR-122 SL and DL P7 neuron isolation after microdissection, bulk RNA-sequencing and support vector machine learning approach to predict SL *vs.* DL neuron position. Right, Prediction of miR-122 single cells using this model. (**C**) Left, schematic diagram of L2/3 and L4 P3 and P7 neuron isolation through birthdate-locked *in utero* electroporation (IUE), single-cell RNA sequencing and support vector machine learning approach to predict P3 *vs.* P7 identity (*ie*. maturation score), (E14-P3, E15.5-P3, E14-P7 and E15.5-P7, n=3 each). Right, maturation and layer identity (ID) scores of P3 and P7 E14 / E15.5 born neurons. (**D**) Passive properties and spontaneous activity recordings in L2/3 scrambled (scram) and miR-122 neurons at P21 (scram, n=26 cell from 3 animals; miR-122, n=22 cells from 3 animals. Passive properties: Ih current, Capacitance, Input resistance, Membrane constant, Sag ratio, Spike threshold, AHP, Rheo-base, Spike peak, Spike ratio, RMP, V_{drop}, V_{min}, V_{end}, Spike delay. (**E**) CASP3 expression in cortex at P7. Quantification of CASP3+ in scram and miR-122 overexpressing cells (scram and miR-122, n=3 each). (**F**) BRN2 expression in L4 neurons in scram and miR-122 conditions (scram and miR-122, n=3 each). (**G**) Left: CUX1 expression in SL neurons in scram and miR-122 conditions (scram and miR-122, n=3). Right: Violin plot of RorB and Cux1 expression in ScRNA-seq of P7 scram and miR-122 neurons elctroporated at E14.5. Data are represented as mean ± SEM (A, E) or mean ± SD (D). (A) Two-way ANOVA; D-E, Unpaired t-Test; One-way ANOVA (F-G). Biological replicates and recorded cells are distin-guished by circles in the bar plots (A and D, respectively). *$p < 0.05$, **$p < 10^{-2}$, ***$p < 10^{-3}$, ****$p < 10^{-4}$.
